# A longitudinal and experimental study of the impact of knowledge on the bases of institutional trust

**DOI:** 10.1371/journal.pone.0175387

**Published:** 2017-04-17

**Authors:** Lisa M. PytlikZillig, Christopher D. Kimbrough, Ellie Shockley, Tess M. S. Neal, Mitchel N. Herian, Joseph A. Hamm, Brian H. Bornstein, Alan J. Tomkins

**Affiliations:** 1 University of Nebraska Public Policy Center, Lincoln, Nebraska, United States of America; 2 University of Nebraska – Lincoln, Department of Psychology, Lincoln, Nebraska, United States of America; 3 School of Criminal Justice & Environmental Science and Policy Program, Michigan State University, East Lansing, Michigan, United States of America; Goethe-Universitat Frankfurt am Main, GERMANY

## Abstract

This study examined a knowledge-centered theory of institutional trust development. In the context of trust in water regulatory institutions, the moderating impact of knowledge was tested to determine if there were longitudinal changes in the bases of institutional trust as a function of increases in knowledge about a target institution. We hypothesized that as people learn about an institution with which they were previously unfamiliar, they begin to form more nuanced perceptions, distinguishing the new institution from other institutions and relying less upon their generalized trust to estimate their trust in that institution. Prior to having specific, differential information about a new institution, we expected institutional trust to be a function of generalized trust variables such as dispositional trust and trust in government. The longitudinal experiment involved 185 college students randomly assigned to one of three information conditions. Every 3 months for 15 months, participants read information about water regulatory institutions or a control institution. At each time point, participants reported their trust in and perceptions of the trust- and distrust-worthiness of the water regulatory institutions. Participants also completed measures of knowledge of water regulatory institutions, dispositional trust, and governmental trust. Our manipulation check indicated that, as expected, those in the experimental group increased in subjective knowledge of water regulatory institutions to a greater extent than those in the control condition. Consistent with our hypotheses, there was some evidence that, compared to the control group, the experimental group relied less on their general trust in government as a basis for their trust in water regulatory institutions. However, contrary to our hypotheses, there was no evidence the experimental group relied less on dispositional trust as a basis for institutional trust. There also was some evidence the experimental group’s trust in water regulatory institutions was less affected by fluctuations of trustworthiness (but not distrustworthiness) perceptions over time. This suggests that knowledge results in the development of more stable institutional trust attitudes, but that trustworthiness and distrustworthiness perceptions may operate somewhat differently when impacting trust in specific institutions.

## 1. Introduction

Trust is essential to societal functioning. From its effects on markets to the operation of democracy, trust in institutions facilitates relationships and permits social exchanges [1–4]. Understanding how people’s trust in an institution (hereafter, referred to as “institutional trust”) develops and evolves over time is not only important in its own right, it is even more significant given evidence that the American public’s trust and confidence in its government has decreased steadily over the past few decades [5–8]. This trend of decreasing trust has been identified by both scholars and policy makers as one of the fundamental problems facing democratic societies today [9].

Although considerable work has examined changes in the public’s institutional trust over time and the factors that influence such changes [10, 11], a number of gaps remain. Despite the potentially critical role that knowledge is conjectured to play in the development of trust in an institution [12, 13], there has been relatively little empirical examination of the impact of information and knowledge on institutional trust [13]. Similarly, research has yet to address systematically how or whether the large number of hypothesized bases that underlie and affect people’s trust in an institution may change over time. Such bases include pre-existing trustor dispositions, assessments of the institution’s worthiness of being trusted, and loyalties or commitments the trustor may form toward the institution [14–17].

A number of theories suggest the most important bases of trust will change as people gain “sophistication”—that is, as they learn more about and/or have experience with the trustee [18–22]. However, there are few longitudinal studies of whether and how these bases change over time and, in particular, as people increase their knowledge about an institution. As others have noted, cross-sectional studies are sufficient to examine different bases of trust, but longitudinal studies are needed to disentangle closely related and potentially reciprocal variables [23–25].

In the present study, we address some of the gaps in the literature by conducting a longitudinal experiment examining institutional trust and its potential bases in individuals who had very little initial knowledge or experience with the target institution. We focus on trust in water regulatory institutions—governmental entities that are unfamiliar to most in the general public, but ones likely to become increasingly important as climate change impacts water availability and quality across the nation [26].

### 1.1. Institutional trust and knowledge

Many definitions of trust exist [27]. We define *institutional trust* as an attitude toward a specific institution (or organization, business, etc.) characterized by positive expectations that the institution will appropriately fulfill its functions [11, 28] (S2 File, Note A). Trust is most often measured with survey/questionnaire items directly assessing the degree of trust/confidence one has in a given institution to do its job (e.g., “how much of the time can you trust [institution] to do what is right?” or “how much confidence do you have in [institution or those running the institution]?”) [15, 29–31]. Institutional trust conceptualized in this way is not especially nuanced and instead addresses an overarching, global assessment of an institution. Most major public opinion polls measure trust in this manner, and it is these measures that primarily fuel the conversation regarding the public’s decreasing institutional trust, although it is possible that a more nuanced understanding of trust might contribute to a more precise understanding of how people are assessing societal institutions (S2 File, Note B).

One factor that appears to influence how people assess institutions is familiarity with the institutions. Research has shown changes in knowledge about an institution can have important effects on attitudes toward the institution. Studies have documented both positive [13] and negative [32] effects of increased knowledge on institutional trust. Knowledge may also lead to the ability to differentiate between specific aspects of—and therefore to hold more nuanced views of—an institution. For example, one study found that greater knowledge about Congress can enable individuals to distinguish between Congress as an institution and individual lawmakers [32].

Thus, there is good reason to expect that knowledge about an institution shapes levels of, and reasons for, individuals’ trust in it (i.e., their institutional trust) [23, 33]. We conceptualize three “stages” of institutional trust that vary in terms of the bases of trust and amount of knowledge of the trustor: (1) generalized trust as the primary basis of both institutional trust and trustworthiness perceptions in the no or low-knowledge *undifferentiated stage*, (2) trustworthiness perceptions as providing shifting bases for institutional trust during a second stage of increasing knowledge and differentiation (*differentiated stage)*, and (3) felt commitment as the basis of institutional trust in the high-knowledge *committed stage* (S2 File, Note C). While our experiment and hypotheses focus primarily on the first two stages, we describe all three stages to provide context.

### 1.2. A Three-stage developmental model of knowledge-based trust

#### 1.2.1. Undifferentiated stage 1: Generalized trust as the source of undifferentiated institutional trust and trustworthiness perceptions

Various forms of *generalized trust*, like the trustor’s propensity to trust people (i.e., dispositional trust) or propensity to trust certain types of institutions (e.g., governmental trust), are typically important predictors of trust in *specific* institutions [18, 23, 34–37]. These generalized trust propensities are similar to personality traits in that they differ between people and likely stem from a combination of genetic predispositions, biological factors, and prior social and emotional experiences with other people and institutions [38]. For instance, a person may have a generally trusting personality and may assume most strangers are well-intentioned. Another person may have an untrusting personality such that the individual is suspicious of most people. Similarly, a person might be prone to trusting a range of institutions even when unfamiliar with them (for example, defaulting to trusting the police, fire fighters, and city council when moving to a new town) or might be prone toward suspicion of most institutions. Generalized trust is thought of as relatively stable; however, like personality traits, it is expected to show development, evolving over time in response to societal and life changes and events [39–42].

Many theories propose that generalized trust (i.e., a propensity to trust *across* targets) and institutional trust (i.e., trust in a *specific* institution) are distinct but are likely to be positively related [14, 18]. However, the variance shared by generalized trust and institutional trust varies, and it is not always important or even statistically significant [22, 23]. Some researchers have thus proposed that a person’s generalized trust provides a “baseline” that is applied when forming judgments of targets or trustees that are not well-known. That is, without specific knowledge about an institution, trustors will be in an “undifferentiated” stage in which their trust attitudes toward that institution are relatively undifferentiated from their generalized trust attitudes.

Similarly, during this stage, there is likely to be a lack of differentiation among institutional trust, generalized trust, and one’s *perceptions of the trustworthiness* of the target institution. *Trustworthiness perceptions* refer to assessments of the target that suggest whether it is, or is not, worthy of trust. These perceptions, unlike institutional trust, are quite nuanced. Past research has identified several perceived trustworthiness constructs (or “facets”) related to institutional trust. Facets of trustworthiness include perceptions of a trustee’s ability, benevolence, integrity [18, 35, 36, 43], and perceived legitimacy [44–47]. Further facets include procedural justice constructs such as whether the target or trustee is perceived as having respect for the trustor, whether the trustee exhibits neutrality, whether the trustee gives voice to stakeholders (such as the trustor), and whether the trustee is characterized by fairness [48–51]. Cynicism—a view that the institution’s motives for acting run counter to the interests of the individual [46]—may represent an explicitly negative aspect of perceived (dis)trustworthiness.

Perceptions of trustworthiness are strong and robust predictors of other trust attitudes [52, 53], even to the point of making statistical and measurement distinctions between “trustworthiness” and direct assessments of “trust” quite difficult [23]. We hypothesize this lack of distinction may be especially true in Stage 1 (undifferentiated), where trustors are likely to ascertain trustworthiness judgements in the same way as they determine their baseline institutional trust—that is, based on their generalized trust. This would result in strong relations between institutional trust and trustworthiness assessments—as well as between different types of trustworthiness assessments (e.g., perceived benevolence versus perceived competence)—because of a lack of institution-specific information.

#### 1.2.2. Differentiation stage 2: Differentiation of trust constructs based on changing perceptions of trustworthiness

We propose that stage 2 occurs when increasing knowledge of the institution leads to differentiation between various facets of perceived trustworthiness, which then leads more generally to differentiation between generalized trust and trustworthiness perceptions and between generalized trust and institutional trust (see H1 and H2 below). That is, trustors gain knowledge and sophistication about an institution, and form more nuanced trustworthiness judgments (e.g., judging the institution as competent but not benevolent, or as benevolent but not honest). This results in, first, the differentiation of trustworthiness judgments (now based on knowledge of the specific institution) from one’s generalized trust; and, second, reduced need to rely on generalized trust to determine one’s institutional trust, because one can rely on one’s more specific (and differentiated) trustworthiness judgments.

These arguments are consistent with prior research and theory. For example, relating to the differentiation of trustworthiness perceptions, Mayer and his colleagues [18] propose that people quickly form impressions of a trustee’s integrity and competence, whereas benevolence assessments may take longer to develop. Schoorman, Mayer, and Davis [54] similarly argued that some trustworthiness perceptions—such as benevolence and integrity judgments—may be too highly correlated to distinguish early on, but differentiation becomes possible with increasing knowledge of and experience with a target. Relating to the differentiation of generalized trust and institutional trust, the sophistication-interaction theory of public opinion [20] proposes that “low sophisticates” with relatively little knowledge of an issue do not have the ability to rely on domain-specific principles to guide their preferences and therefore must draw upon more general concerns to a greater extent than “high-sophisticates” [55–57]. Although primarily applied to policy preferences, when extended to the area of trust attitudes, this theoretical perspective suggests those who have low knowledge of an institution may rely on heuristic cues or shortcuts—such as relying on their sense of generalized trust—to estimate how much they should trust that institution in lieu of specific information. In contrast, individuals who are more knowledgeable may rely less upon generalized trust because they can draw on specific knowledge of the institution and its role in the governing process and to make judgments about specific aspects of its trustworthiness. In support of this theoretical extension, some data have shown that, as the trustor gains information about the trustee, the influence of generalized trust on trust in specific targets decreases in favor of more specific evaluations [18, 57, 58].

Finally, our knowledge-centered theory of institutional trust development proposes that the differentiation of trustworthiness facets also has implications for their specific relationships to institutional trust. Depending on the context, we theorize that changes in certain trustworthiness judgments (instead of generalized trust) will become the source of changes in individual’s trust in that institution (institutional trust). However, as trustworthiness judgments become more differentiated from each other and from generalized trust factors, some trustworthiness factors may be more important predictors than others (see H3 below). The precise pattern of differences, however, is difficult to predict given the current state of the literature [59].

#### 1.2.3. Committed stage 3: Committed institutional trust (or distrust) characterized by increasing stability

*Commitment* often refers to a sense of loyalty to an institution that is resilient in the face of specific dissatisfactions encountered over time [15, 60–63]. However, commitments could be either positive or negative—with negative commitments characterized by “enduring distrust” or stable resistance rather than loyalty [64, 65]. Commitment, therefore, reflects an increasingly stable and persistent feeling toward a specific institution that differs among individuals. Commitment also typically is viewed as arising from continued and repeated exposure to the institution over time rather than acute interactions at a single point in time [66, 67].

Commitment has been examined as both a basis and outcome of trust [15, 19, 68]. Here, we propose the development of committed trust (or distrust) attitudes will be reflected in greater attitude stability over time. Thus, although time-specific variations in trustworthiness perceptions will affect trust, as more time passes and more knowledge of an institution is gained, one’s level of institutional trust will gradually stabilize and become less dependent upon fluctuations in one’s perceptions of the institution’s trustworthiness [68, 69] (see H4 below). Consistent with this hypothesis, theories of attitude development suggest that as people gain knowledge, their attitudes become more stable and less influenced by additional information [70, 71].

### 1.3. Summary of hypotheses

Based on our knowledge-centered theory of institutional trust development, we developed four hypotheses that were tested in the current study. The first two hypotheses relate to the differentiation of generalized trust and other trust-relevant constructs as participants move from stage 1 to 2, and thus are very similar:

(H1, H2) As knowledge about a specific institution increases, the relationships will decrease between *generalized trust* variables (e.g., dispositional trust and non-specific governmental trust) and both
   (H1) *institutional trust* (i.e., trust in a *specific* institution), and
   (H2) *trustworthiness* variables (i.e., perceptions of a *specific* institution’s trust-relevant *qualities* such as its benevolence, integrity, and competence).

Our third hypothesis also focuses on movement from stage 1 to 2, but focuses on the relations between institutional trust and trustworthiness perceptions as people form more nuanced and knowledge-based views, and as specific facets of trustworthiness become distinguished from one another:

(H3) As knowledge of a specific institution increases, the relationships between institution-specific *trustworthiness* variables and *institutional trust* (trust in that specific institution) will *change*.

This hypothesis is not directional—as noted in our theoretical description, the direction of change is uncertain and may be context-specific.

Finally, although there is little prior research to inform how long it might take for commitment to form toward an institution, we nonetheless posed a fourth hypothesis that addressed whether there was evidence of stage 3 processes (i.e., attitude stability) in our data:

(H4) Among those gaining in knowledge, over time, changes in institutional trust will become less associated with fluctuations in trustworthiness perceptions.

### 1.4. The current study

To test our four hypotheses, we conducted a longitudinal experiment in which every 3 months for 15 months we repeatedly assessed all participants’ institutional trust and trustworthiness perceptions of water regulatory institutions. In addition, participants read information about an institution every three months. Participants were randomly assigned to one of three information conditions, two of them were experimental conditions and one was a control condition. Participants in the experimental conditions read information about water regulatory institutions, while participants in the control condition read about a non-water-related institution. Initial analyses indicated our sample had very little prior knowledge of either the experimental or control institutions. The control condition exposed participants to the same longitudinal process in which they completed the same repeated measures and learned about *an* institution, but the institution they learned about was not relevant to the target institution that *all* participants repeatedly evaluated. This means that participants in the control condition were reporting their institutional trust in and trustworthiness perceptions of an institution they had *not* been learning about during this study.

This design allowed us to assess change in our trust-relevant variables over time, as well as whether and how relationships among the variables changed. We examined the effects of knowledge gains about an institution in two ways. First, participants either learned about the target institution (experimental group), or they did not (control group). Second, among those who did learn about the target institutions, we examined this learning process longitudinally, assuming at the outset that knowledge should positively correlate with time.

## 2. Method

### 2.1. Ethics and human subjects

All participants in this research were age 17 or older and provided written consent to participate. The research met the requirements for exemption from the need to obtain parental consent for those participants who were not of legal age of majority (which is age 19 in NE); thus, no consent was obtained from parent/guardians of those participants. All aspects of this research, including the consent procedure and waiver of parental consent, were presented to and approved by the University of Nebraska-Lincoln Institutional Review Board for the Ethical Treatment of Human Subjects (IRB Approval #: 20101211178EP). Six surveys were administered to 202 students from two Midwestern universities. The first survey asked participants to complete baseline measures, and the experimental manipulation started on the second survey. Consequently, we removed participants completing only the first survey (S2 File, Note D). The final set of 185 participants averaged 20.76 years of age (*SD* = 3.39); 58% self-identified as women and 95% as White.

### 2.2. Procedure

Participants were recruited via email and classroom visits by the authors from a variety of science classes (e.g., biology, environmental science, psychology) (S2 File, Note E). Participants completed 6 surveys over 15 months, with approximately 3 months between surveys. We emailed participants a link to each online survey to complete at their convenience during a two-week period and sent reminder emails. To provide incentive against attrition, participants received increasing payments for each survey they completed, with a total payment of $155 if all surveys were completed.

In each survey, participants completed a battery of questions including measures of our key variables of dispositional trust, institutional trust in and trustworthiness perceptions of the water institutions (the target/trustee of interest in this study), and subjective and objective knowledge of the water regulatory institutions. All participants (control and experimental) completed the same battery of measures in the surveys administered after the randomly assigned information was presented.

### 2.3. Materials and measures

#### 2.3.1. Information manipulation

We designed the information manipulations to enhance participant knowledge of the institution to which the participant was randomly assigned. The institutions were two Nebraska water-regulatory agencies, Department of Natural Resources (DNR) and Natural Resource Districts (NRD), and the state’s child welfare department, Health and Human Services (DHHS). The first information exposure presented basic descriptive information, including the responsibilities, jurisdictions, and authority of the relevant institution. The information provided during subsequent contacts was comprised of various newspaper articles and factsheets tailored to address specific topics. The information in the water-regulatory institution conditions was always relevant to Nebraska’s water regulation policies. The information in the control condition was always related to Nebraska’s child welfare policies. To help ensure knowledge gains, information about each institution was presented along with questions designed to encourage active engagement (e.g., “can you think of any instances in which you, or someone you know, might have been affected by the *[institution’s]* decisions?”). We also included reading-check questions that were specific to the reading and often drew attention to factors that would be relevant for trust judgments (e.g., “The current drought has resulted in closing notices being sent by the DNR to surface water irrigators. What method is being used in deciding priority for irrigation?”).

Although we began with three institution information conditions, a preliminary review of participant comments suggested participants might not be distinguishing between the two water-regulatory institutions. Because the two institutions do, in fact, serve overlapping roles (e.g., both are major players within the framework for setting allocations for irrigation), we examined whether it was appropriate to combine the DNR and NRD conditions and measures. Bivariate correlations between measures of institutional trust in the two water regulatory institutions were strong at each time point, with Pearson correlation coefficients ranging from .70 to .92, all *p*’s < .01. Correlations between trustworthiness ratings of the two water institutions (e.g., competence, legitimacy, etc.) were also high at each time point, ranging from .85 to .97, all *p*’s < .01. In addition, bivariate correlations between subjective knowledge ratings for the DNR, NRD, and general water regulation were significant within time points, ranging from .55 to .77, all *p*’s < .01.

Given the lack of statistical distinction between the two water regulatory institutions and their factual overlap, we combined the two conditions. Our experimental variable was therefore dichotomously coded: 0 = control information (DHHS), 1 = experimental information (DNR or NRD).

#### 2.3.2. Measures

The following items were completed by each participant at each time point (the primary measures and items are in the S1 File, Appendix of Measures, in the online Supporting Information). Table 1 contains the descriptive statistics for each scale as measured at Survey 1, as well as the range of internal reliabilities obtained at each of the six surveys. Unless otherwise noted, participants responded to items in our measures by rating their agreement on a 7-point Likert scale ranging from strongly disagree (coded 1) to strongly agree (coded 7, with a neutral midpoint = 4), and items were averaged (after reverse-scoring if appropriate) to create scales.

**Table 1:** Descriptive statistics for institutional trust and trustworthiness scales at survey 1 (T0) and internal reliability across time points (T0 to T5).

Measure	Mean (T0)	SD (T0)	Cronbach’s α ranges (T0-T5) Low to High	
Knowledge				
Subjective Knowledge	0.568	0.624	.832	.893
Objective Knowledge	3.692	2.744	.543	.861
Dependent Variable				
Institutional Trust	4.996	0.836	.927	.958
Positive Trustworthiness	4.918	0.715	.978	.985
Legitimacy	5.029	0.760	.880	.943
Respect	5.008	0.811	.901	.937
Bias	3.838	0.696	.740	.880
Voice	4.805	0.740	.802	.902
Distributive Justice	4.992	0.857	.897	.944
Loyalty	4.770	0.784	.846	.878
Shared Values	4.748	0.836	.908	.945
Benevolence	4.905	0.785	.836	.929
Competence	5.034	0.855	.868	.916
Honesty/Integrity	4.894	0.813	.886	.949
Distrustworthiness	3.688	0.689	.854	.919
Cynicism	3.538	0.796	.784	.891
Obligation to Obey	5.000	0.859	.856	.914
Generalized Trust				
Governmental Trust	4.948	0.961	.846	.898
Dispositional Trust	5.232	0.805	.894	.935

*Notes*. T0 = time zero and survey 1, T5 = time 5 and survey 6. Subjective knowledge was on a scale from 0 (low)-4 (high); objective knowledge reflects the total number of correct answers to 12 factual questions (possible range of 0–12). The remainder of the scales could range from 1 to 7 as described in the text.

**Knowledge****:** Like past research investigating political sophistication [72–75], we included both objective and subjective knowledge measures as checks of our information manipulations. All participants (both control and experimental) indicated their *subjective knowledge* of each of the two water regulatory institutions by responding to the item: “How knowledgeable are you about the [Institution]?” (response options: 0 = not at all, 1 = slightly, 2 = moderately, 3 = very, and 4 = extremely). We also asked a similar question about “water regulation in general.” We averaged across the items for the two water regulatory institutions and water institutions in general to create a three-item subjective knowledge scale (Cronbach’s α = .83-.89 across the six time points). To assess *objective knowledge* of water regulatory institutions, all participants also answered 12 factual multiple-choice questions in each survey concerning the specific water regulatory institutions and water regulation in general. The total number of correct responses was used to indicate participant objective knowledge at each survey (S2 File, Note F). All questions were created specifically for this study and had four possible responses, with one correct answer. As shown in Table 1, subjective and objective knowledge at the first survey was very low.

**Institutional trust****:** Our institutional trust scale consisted of four items that represented direct reports of trust in the specific target institution (i.e., water regulatory institutions), without specifying reasons for that trust (e.g., “My confidence in the [institution] is high”). As previously noted, it is often unclear exactly what such items are measuring [76], but they are important because they are commonly used to assess trust in institutions [15, 29–31], especially in large national and international surveys.

**Trustworthiness perceptions****:** Our trustworthiness items assessed trust-relevant constructs that represent potential *bases* or *reasons* for psychological trust (e.g., “[institution] is honest”). We term these constructs “trustworthiness perceptions,” because most of the constructs are perceptions of a specific institution that make that institution “worthy” of trust and may be viewed as antecedents or bases for one’s direct expression of trust [18]. All participants rated Nebraska’s water regulatory institutions on 40 items representing 12 different trustworthiness constructs, including perceptions of competence (2 items), benevolence (3 items), and integrity (3 items), corresponding to constructs in the Mayer, Davis, and Schoorman [18] model, as well as other commonly studied constructs [77]: perceptions of shared values (3 items) [78]; perceived legitimacy (4 items), cynical perceptions (4 items), and felt obligation to obey (4 items) [61]; loyalty (4 items) [15]; and justice constructs of perceived respectfulness (3 items), bias (4 items), voice (3 items), and distributive justice (3 items) [79–81]. Items assessing these constructs were taken directly or adapted from a variety of prior measures [16, 57, 82] and, upon evaluation (S2 File, Note G), were used to form two scales assessing *distrustworthiness* perceptions (2 scales, bias and cynicism, comprised of 8 items total), and *positive trustworthiness* (the remaining 10 scales, comprised of 32 items total).

**Generalized trust****:** Finally, participants responded to 9 items pertaining to their dispositional trust, and 7 items to assess trust in governmental institutions generally (S2 File, Note H).

### 2.4. Analytic strategy

#### 2.4.1. Manipulation checks

To confirm the effect of our institutional knowledge manipulations, we examined whether longitudinal change in subjective and objective knowledge (about water regulatory institutions) across the six surveys was a function of time and experimental condition using multilevel general linear regression in SAS 9.3 PROC MIXED, with restricted maximum likelihood estimation. Predictors included a random intercept, a fixed effect of institution manipulation (coded 0 = control, 1 = experimental), fixed and random effects of time (coded as 0–5), and the interaction between time and the institution manipulation.

#### 2.4.2. Preliminary analyses

We next examined overall between-participant differences in our primary measures (institutional trust, positive trustworthiness, distrustworthiness, trust in government, and dispositional trust) by estimating five random intercept, unconditional means models (also known as null or empty models) which partitioned variance into within- and between-participant components for each measure [83]. We then examined baseline ratings as well as linear change in each primary measure across surveys by estimating unconditional longitudinal models including random and fixed effects of intercept and linear time slope. These models allowed us to test for significant between-participant variance in the effects of time (coded 0, 1, 2, 3, 4, and 5) across participants and to establish estimates of intercepts and their variances. Finally, using the best-fitting unconditional models, we added the effect of the institution information manipulation to each model to examine whether that effect was significant within each univariate model, and whether it impacted other results from each model.

#### 2.4.3. Hypothesis testing

We tested Hypotheses 1 through 4 in two different ways. First, we used multivariate multilevel modeling (MMLM). This method is appropriate when there is non-zero variation in dependent and independent variables’ change over time, and provides several advantages over univariate, multilevel modeling, including more powerful tests of fixed effects and a reduction of Type I error [83–85]. In our MMLM models, we examined the within-participant relations between generalized trust measures and institutional trust and the within-participant relations between perceptions of institutional trustworthiness or distrustworthiness and institutional trust. Predictors included manipulation (coded 0 = control, 1 = experimental) as well as the predictors in the best-fitting univariate models for each variable. Our hypotheses predicted that the within-participant correlations between residuals involving generalized trust and both trustworthiness variables and institutional trust would be stronger for control group than experimental group participants. We also predicted the correlations between trustworthiness (i.e., positive trustworthiness and distrustworthiness perceptions) and institutional trust would differ between groups. For these MMLM models, we utilized PROC GLIMMIX with restricted maximum likelihood estimation and examined specific hypotheses using ESTIMATE, CONTRAST, and COVTEST statements.

Initial multivariate analyses on the larger models indicated the data did not contain enough estimable variability between participants to attribute to random effects. Due to this limitation, additional models were estimated using a “slopes-as-outcomes” approach [83]. This approach predicts institutional trust by estimating grand mean-centered individual intercepts and linear change slopes of predictor measures as between-participant (BP) predictors, and time-varying residuals as time-varying within-participant (WP) predictors. In each model, we examined the time × manipulation × BP predictors, as well as the time × manipulation × WP predictors interactions, to determine if the effects of the BP or WP predictors of institutional trust changed over time differently between the control and experimental groups. Significant interactions involving manipulation and predictor slopes would indicate differences between groups in how change over time in a predictor related to change over time of the outcome variable. For example, significant interactions involving time, manipulation, and WP variance in a predictor would indicate differences between groups in how deviation from one’s predicted value on the predictor relates to the outcome variable over time. Our theoretical model predicted closer relations among the control group that remain relatively constant over time and, in general, decreasing relations over time for the experimental group (for H1, H2, and H4; H3 was nondirectional). (Note that the longitudinal data, formatting, and syntax for all models can be found in S3, S4 and S5 Files, respectively).

## 3. Results

### 3.1. Manipulation checks

**Fig 1:**
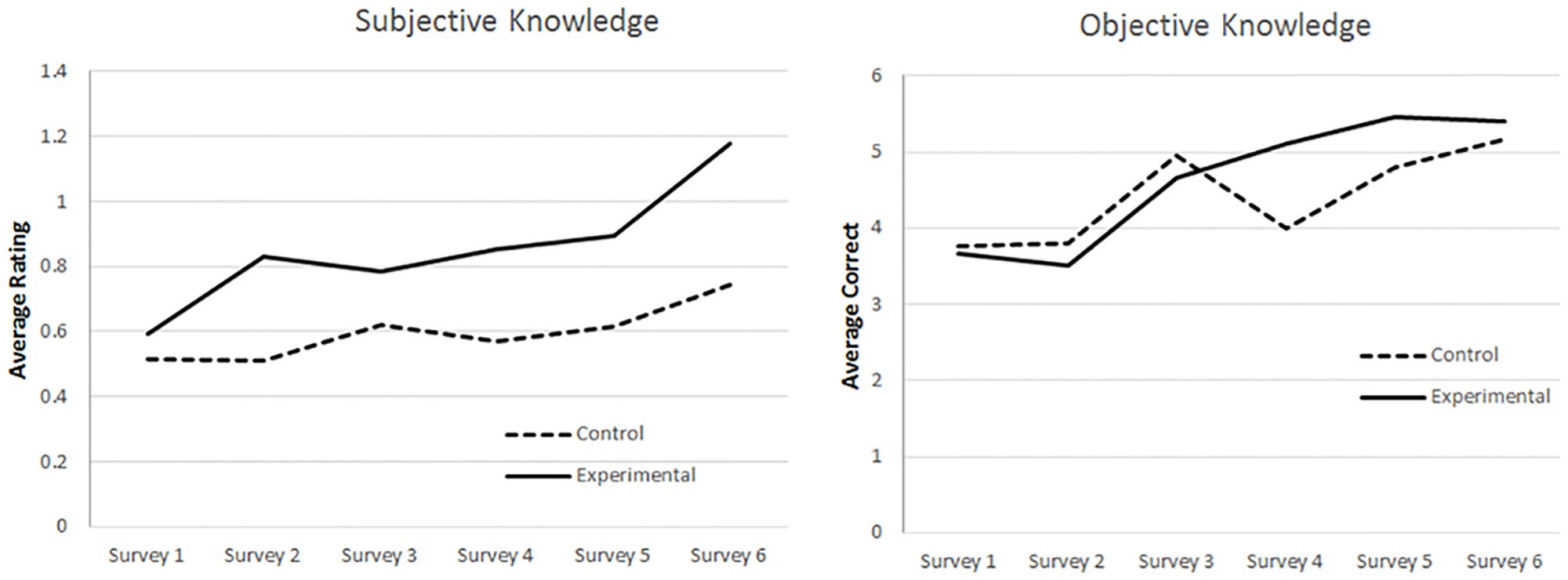
Average levels of subjective and objective knowledge over time for the experimental and control groups. *Notes*. Subjective knowledge (left panel) was on a scale from 0 (low)-4 (high); objective knowledge (right panel) reflects the total number of correct answers to 12 factual questions (range 0–12). Preliminary manipulation checks indicated the increases in subjective knowledge differed between the experimental and control groups as expected. The difference in objective knowledge increases was in the predicted direction but not statistically significant.

As can be seen in Table 2 and Fig 1, our manipulation appeared to influence both knowledge types, though the time x manipulation interaction was significant only for subjective knowledge. The non-significant effect of manipulation on the intercept at survey 1 (time = 0; *B*s = .13, -.28; *SEs* = .09, .38 *Fs*(1,184; 1, 182) = 2.09, .54; *ps* = .15, .46, for subjective and objective knowledge respectively) confirms that average subjective and objective knowledge ratings did not differ between conditions prior to the first institution manipulation (S2 File, Note I). Although subjective knowledge mean scores increased significantly for those in the control condition (i.e., *B*_time_ = .04, *p* < .001), the significant time × institution interaction indicated that subjective knowledge increased at a greater rate for those in the water regulatory experimental condition (interaction effect *B* = .06, *SE* = .02, *p* = .01), becoming significantly different at survey 2 (time 1, *t*(186) = 2.25, *p* = .03), and remaining so for the remainder of the study. For objective knowledge scores, the difference (i.e., time × institution interaction) was in the expected direction but was not statistically significant.

**Table 2:** Parameter estimates for manipulation check models predicting knowledge.

**Subjective Knowledge**					
**Fixed Effects**	B	*SE* B	*df*	*F*-value	*p*-value
Constant	0.496	0.075	N/A	N/A	N/A
Time (Linear Slope)	0.039	0.019	1, 147	37.28	< .001
Information Manipulation	0.132	0.091	1, 184	2.09	.150
Time (Linear Slope) × Information Manipulation	0.059	0.023	1, 147	6.90	.010
**Variance Parameters**	Estimate	*SE*	*Z*-value	*p*-value	
Random Intercept	0.252	0.036	7.01	< .001	
Time (Linear Slope) Variance	0.006	0.002	3.02	.001	
Intercept / Slope Covariance	-0.004	0.007	0.66	.506	
Residual Variance	0.159	0.010	16.70	< .001	
**Objective Knowledge**					
**Fixed Effects**	B	*SE* B	*df*	*F*-value	*p*-value
Constant	3.796	0.313	N/A	N/A	N/A
Time (Linear Slope)	0.267	0.085	1, 149	44.03	< .001
Information Manipulation	-0.282	0.382	1, 182	0.54	.461
Time (Linear Slope) × Information Manipulation	0.153	0.104	1, 149	2.19	.141
**Variance Parameters**	Estimate	*SE*	*Z*-value	*p*-value	
Random Intercept	4.400	0.631	6.97	< .001	
Time (Linear Slope) Variance	0.189	0.047	4.05	< .001	
Intercept / Slope Covariance	-0.668	0.151	4.43	< .001	
Residual Variance	2.691	0.167	16.08	< .001	

*Notes*. Model coding was as follows: Time: 0 = First survey (reference group), 1 = Second survey, 2 = Third survey, etc.; Institution: 0 = DHHS (reference/control group) information, 1 = DNR or NRD information. B = Unstandardized parameter estimate. SE = standard error. Each estimate indicates the effect of each condition compared to the reference group, as estimated by the model.

### 3.2. Preliminary analyses

#### 3.2.1. Correlations

Table 3 displays the means, standard deviations, and a correlation heat map plot (in which darker shading indicates stronger correlations) to illustrate the varied strength of correlations for study variables at each survey. Consistent with prior studies [16], very high correlations (*r*s > .90) were observed between unspecified institutional trust and trustworthiness at each time point. As expected, the lowest correlations were those among the generalized trust variables (dispositional trust and governmental trust) and specific trust variables (institutional trust and trustworthiness perceptions).

**Table 3:** Correlations among study variables.

			**Institutional Trust**						**Trustworthiness**						**Distrustworthiness**		
**Variable**	**M**	**SD**	1	2	3	4	5	6	7	8	9	10	11	12	13	14	15
1. Inst. Trust, S1	5.00	0.84															
2. Inst. Trust, S2	5.10	0.80	.69														
3. Inst. Trust, S3	5.20	0.82	.66	.67													
4. Inst. Trust, S4	5.25	0.81	.66	.74	.72												
5. Inst. Trust, S5	5.22	0.89	.57	.56	.66	.79											
6. Inst. Trust, S6	5.28	0.82	.55	.64	.73	.78	.77										
7. Trustworth., S1	4.92	0.72	.94	.70	.66	.63	.56	.55									
8. Trustworth., S2	5.00	0.71	.70	.94	.68	.73	.59	.67	.74								
9. Trustworth., S3	5.11	0.74	.65	.66	.95	.72	.67	.71	.67	.71							
10. Trustworth., S4	5.17	0.72	.63	.71	.73	.92	.79	.79	.67	.77	.78						
11. Trustworth., S5	5.16	0.80	.56	.52	.67	.75	.95	.73	.59	.59	.73	.80					
12. Trustworth., S6	5.20	0.71	.54	.60	.71	.74	.76	.93	.59	.68	.74	.81	.78				
13. Distrustw., S1	3.69	0.69	-.62	-.51	-.46	-.49	-.46	-.41	-.60	-.49	-.47	-.49	-.46	-.42			
14. Distrustw., S2	3.60	0.69	-.56	-.68	-.50	-.55	-.42	-.50	-.61	-.69	-.52	-.56	-.41	-.49	.61		
15. Distrustw., S3	3.50	0.78	-.61	-.62	-.66	-.62	-.64	-.62	-.64	-.66	-.67	-.66	-.67	-.64	.57	.69	
16. Distrustw., S4	3.36	0.82	-.60	-.64	-.61	-.72	-.60	-.62	-.64	-.66	-.65	-.74	-.62	-.63	.52	.64	.70
17. Distrustw., S5	3.38	0.88	-.56	-.51	-.57	-.64	-.63	-.58	-.60	-.55	-.61	-.67	-.62	-.59	.54	.61	.71
18. Distrustw., S6	3.29	0.83	-.47	-.55	-.58	-.60	-.56	-.73	-.52	-.60	-.58	-.65	-.58	-.73	.38	.55	.66
19. Disp. Trust, S1	5.23	0.81	.45	.38	.50	.38	.35	.36	.47	.38	.46	.40	.37	.36	-.36	-.25	-.38
20. Disp. Trust, S2	5.25	0.73	.39	.43	.46	.41	.34	.32	.40	.40	.43	.42	.34	.37	-.32	-.27	-.34
21. Disp. Trust, S3	5.41	0.79	.39	.33	.52	.41	.38	.33	.38	.35	.51	.42	.41	.34	-.31	-.23	-.37
22. Disp. Trust, S4	5.37	0.76	.30	.37	.46	.39	.37	.39	.31	.37	.43	.42	.38	.37	-.33	-.22	-.30
23. Disp. Trust, S5	5.38	0.74	.39	.35	.49	.39	.52	.49	.39	.34	.49	.41	.52	.50	-.37	-.21	-.36
24. Disp. Trust, S6	5.42	0.83	.28	.21	.33	.37	.39	.38	.30	.30	.30	.40	.39	.41	-.18^+^	-.17^+^	-.31
25. Govt. Trust, S1	4.95	0.96	.50	.37	.46	.49	.42	.35	.49	.37	.41	.46	.42	.35	-.31	-.27	-.32
26. Govt. Trust, S2	4.90	1.07	.39	.44	.51	.51	.45	.46	.37	.44	.48	.51	.44	.45	-.28	-.31	-.33
27. Govt. Trust, S3	4.88	1.15	.41	.38	.57	.54	.56	.60	.38	.40	.54	.55	.56	.58	-.24	-.30	-.34
28. Govt. Trust, S4	5.17	1.04	.39	.40	.57	.51	.51	.51	.38	.40	.52	.49	.51	.48	-.27	-.26	-.36
29. Govt. Trust, S5	4.90	1.06	.36	.31	.55	.52	.55	.55	.34	.34	.49	.48	.57	.52	-.25	-.30	-.42
30. Govt. Trust, S6	4.97	1.07	.27	.29	.44	.36	.39	.51	.27	.28	.39	.35	.38	.51	-.18	-.31	-.33

			**Distrustworthiness**			**Dispositional Trust**						**Governmental Trust**					
			16	17	18	19	20	21	22	23	24	25	26	27	28	29	30
17. Distrustw., S5			.76														
18. Distrustw., S6			.75	.67													
19. Disp. Trust, S1			-.32	-.22	-.29												
20. Disp. Trust, S2			-.29	-.26	-.29	.71											
21. Disp. Trust, S3			-.34	-.27	-.32	.70	.67										
22. Disp. Trust, S4			-.24	-.29	-.34	.63	.57	.65									
23. Disp. Trust, S5			-.29	-.29	-.35	.64	.71	.67	.62								
24. Disp. Trust, S6			-.23	-.25	-.23	.50	.45	.59	.53	.46							
25. Govt. Trust, S1			-.35	-.37	-.27	.50	.40	.42	.31	.29	.31						
26. Govt. Trust, S2			-.38	-.33	-.33	.29	.49	.31	.18	.38	.23	.58					
27. Govt. Trust, S3			-.37	-.36	-.35	.34	.39	.39	.28	.44	.25	.57	.78				
28. Govt. Trust, S4			-.32	-.36	-.38	.39	.43	.47	.45	.39	.26	.60	.57	.74			
29. Govt. Trust, S5			-.34	-.33	-.38	.31	.41	.41	.29	.46	.34	.51	.69	.77	.77		
30. Govt. Trust, S6			-.27	-.29	-.37	.27	.37	.26	.26	.42	.20	.41	.61	.70	.70	.79	

^+^Correlation significant at *p* < .10 (all other correlations are significant at *p* < .05). Darker shading indicates stronger relationships between variables. S1-S6 refers to survey 1 to survey 6. Correlations between time adjacent repeated measures are in boxes.

#### 3.2.2. Unconditional models

Examination of the unconditional empty models indicated significant between-participant variance for all measures (all χ^2^ (1) > 436.48, *p*’s < .01). Intraclass correlations indicated that the percentage of the total variation residing between participants was 66% for institutional trust, 68% for trustworthiness, 59% for distrustworthiness, 62% for governmental trust, and 60% for dispositional trust.

When examining and modeling the effects of time for each of our main variables (S2 File, Note J), the best-fitting baseline-intercept models for institutional trust and perceived positive trustworthiness each included significant linear and quadratic effects of Time. Both measures showed an overall average significant increase over time (effect of time, linear slope) that gradually became less positive with each subsequent survey (time, quadratic slope)—that is, both institutional trust and perceptions of trustworthiness first increased and then plateaued over time. Thus, by the final survey, the linear effect of time was non-significant for both institutional trust (*estimated linear slope at Survey 6* = -0.02, *t*(709) = 0.60, *p* = .55) as well as positive trustworthiness (*estimated linear slope at Survey 6* = -0.013, *t*(708) = 0.46, *p* = .65). Dispositional trust and perceived distrustworthiness only included a linear effect of time, with dispositional trust showing an overall average significant increase over time and perceived distrustworthiness showing an overall decrease. Finally, the linear effect of time was not significant for governmental trust; thus, on average, trust in government neither increased nor decreased.

#### 3.2.3. Univariate models with information manipulation

**Fig 2:**
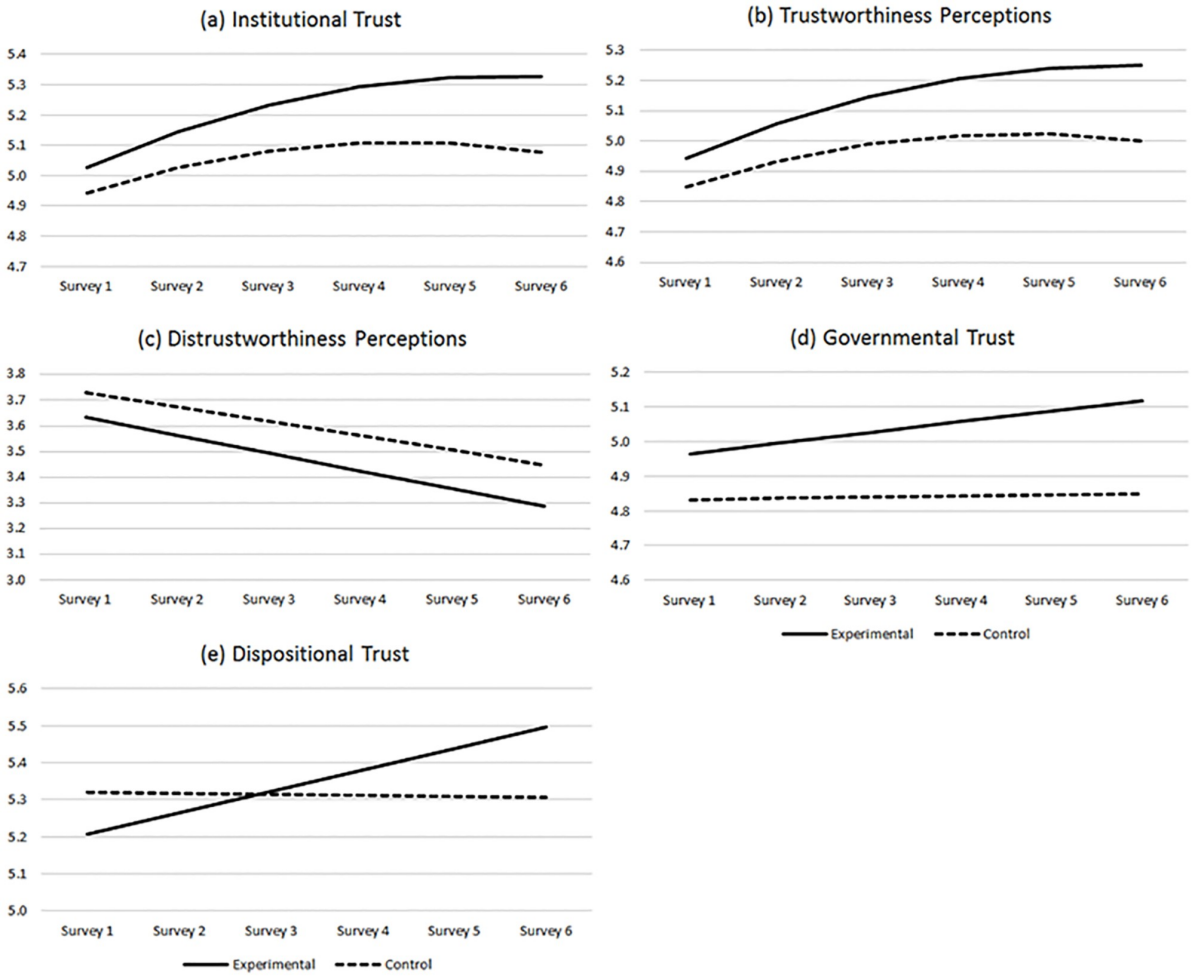
Predicted levels of key variables over time for the control and experimental groups. *Notes*. Predicted values were based on univariate models including time and information manipulation conditions as described in the text. There were no significant differences in the trajectories of the control and experimental groups in Figs 2a-2d. There was a significant difference in slopes for Fig 2e.

The addition of institution manipulation as a predictor resulted in the same overall patterns of change in our primary measures, and the random variances in intercepts and slopes (change over time) remained significant (see Table 4 and Fig 2a–2e). Taken together, these results indicate significant between-participant variance in both the intercepts and change over time for each of our variables.

**Table 4:** Multilevel univariate models for primary outcome measures predicted by information manipulation.

Effect	Estimate	SE	*95% CI*	*DF*	*F/Z*-value	*p-*value
**Institutional Trust**						
Fixed						
Intercept (Survey 1)	4.941	0.100	3.515–6.367	194	N/A	N/A
Time (Linear Slope)	0.098***	0.036	-0.193–0.389	705	13.39	< .001
Time × Time (Quadratic Slope)	-0.014*	0.006		602	5.70	.017
Manipulation effect	0.085	0.121		183	0.49	.484
Time × Manipulation effect	0.034	0.027		143	1.49	.224
Random						
Intercept Variance (Survey 1)						
Control	0.529***	0.115			4.61	< .001
Experimental	0.467***	0.074			6.32	< .001
Time (Linear Slope) Variance						
Control	0.022**	0.007			3.02	.001
Experimental	0.009**	0.003			2.66	.004
Intercept / Linear Slope Covariance						
Control	-0.020	0.022			0.93	.350
Experimental	-0.023	0.012			1.86	.062
Residual Variance						
Control	0.168***	0.018			9.42	< .001
Experimental	0.194***	0.014			13.61	< .001
**Positive Trustworthiness**						
Fixed						
Intercept	4.850	0.088	3.615–6.085	193	N/A	N/A
Time (Linear Slope)	0.096***	0.030	0.737–1.183	704	17.83	< .001
Time × Time (Quadratic Slope)	-0.013**	0.005		601	6.84	.009
Manipulation effect	0.093	0.106		183	0.77	.381
Time × Manipulation effect	0.031	0.023		142	1.83	.179
Random						
Intercept Variance (Survey 1)						
Control	0.397***	0.087			4.57	< .001
Experimental	0.379***	0.058			6.54	< .001
Time (Linear Slope) Variance						
Control	0.013**	0.010			2.54	.006
Experimental	0.008***	0.002			3.14	< .001
Intercept / Linear Slope Covariance						
Control	-0.008	0.015			0.55	.579
Experimental	-0.019*	0.009			1.99	.047
Residual Variance						
Control	0.134***	0.014			9.36	< .001
Experimental	0.131***	0.010			13.59	< .001
**Distrustworthiness**						
Fixed						
Intercept	3.732	0.082	2.827–4.637	183	N/A	N/A
Time Linear Slope	-0.056***	0.024	-0.262–0.150	144	19.09	< .001
Manipulation effect	-0.098	0.101		182	0.95	.331
Time × Manipulation effect	-0.013	0.029		144	0.20	.654
Random						
Intercept Variance (Survey 1)						
Control	0.213***	0.061			3.53	< .001
Experimental	0.354***	0.059			6.02	< .001
Time (Linear Slope) Variance						
Control	0.011*	0.001			1.98	.024
Experimental	0.017***	0.004			3.92	< .001
Intercept / Linear Slope Covariance						
Control	0.011	0.014			0.75	.456
Experimental	-0.008	0.012			0.68	.499
Residual Variance						
Control	0.186***	0.020			9.32	< .001
Experimental	0.183***	0.013			13.57	< .001
**Dispositional Trust**						
Fixed						
Intercept	5.320	0.096	4.139–6.501	184	N/A	N/A
Time (Linear Slope)	-0.003*	0.020	-0.218–0.212	144	5.00	.027
Manipulation effect	-0.111	0.117		183	0.90	.344
Time × Manipulation effect	0.060*	0.025		144	6.05	.015
Random						
Intercept Variance (Survey 1)						
Control	0.363***	0.092			3.96	< .001
Experimental	0.467***	0.075			6.20	< .001
Time (Linear Slope) Variance						
Control	0.012*	0.006			2.14	.016
Experimental	0.002	0.003			0.86	.196
Intercept / Linear Slope Covariance						
Control	-0.017	0.018			0.90	.370
Experimental	-0.023	0.011			2.12	.034
Residual Variance						
Control	0.230***	0.024			9.64	< .001
Experimental	0.212***	0.016			13.55	< .001
**Governmental Trust**						
Fixed						
Intercept	4.833	0.121	2.900–6.766	185	N/A	N/A
Time (Linear Slope)	0.003	0.030	-0.364–0.370	147	0.89	.348
Manipulation effect	0.133	0.148		184	0.80	.371
Time × Manipulation effect	0.028	0.036		145	0.59	.445
Random						
Intercept Variance (Survey 1)						
Control	0.973***	0.213			4.56	< .001
Experimental	0.544***	0.097			5.60	< .001
Time (Linear Slope) Variance						
Control	0.035**	0.012			2.55	.002
Experimental	0.011*	0.005			2.06	.020
Intercept / Linear Slope Covariance						
Control	-0.024	0.038			0.62	.534
Experimental	-0.013	0.018			0.74	.460
Residual Variance						
Control	0.327***	0.035			9.46	< .001
Experimental	0.365***	0.027			13.52	< .001

*p < .05,

**p < .01,

***p < .001.

Model coding was as follows: Time Linear Slope: 0 = First survey, 1 = Second survey, 2 = Third survey, etc.; Manipulation effect: 0 = DHHS (reference/control group) information, 1 = DNR or NRD information. Estimate = Unstandardized parameter estimate. All numerator *DF* = 1. Each estimate indicates the effect of each condition compared to the reference group, as estimated by the model.

There were no significant differences in the effect of time (linear and sometimes also quadratic slopes) between manipulation conditions for institutional trust (Fig 2a), perceived trustworthiness (Fig 2b), perceived distrustworthiness (Fig 2c), or governmental trust (Fig 2d), indicating similar changes over time for both groups on these variables. There was, however, a significant interaction between time and institution manipulation when predicting dispositional trust (Fig 2e). This interaction, which we had not hypothesized, indicated that dispositional trust decreased significantly over time for those in the control condition (time, linear slope = -.003, *p* = .03), while those in the experimental condition significantly increased (time, linear slope = 0.06, *p* < .01).

### 3.3. Hypothesis testing: Multivariate multilevel and slopes-as-outcomes models

#### 3.3.1. Hypothesis 1: Generalized trust predicting institutional trust

**Fig 3:**
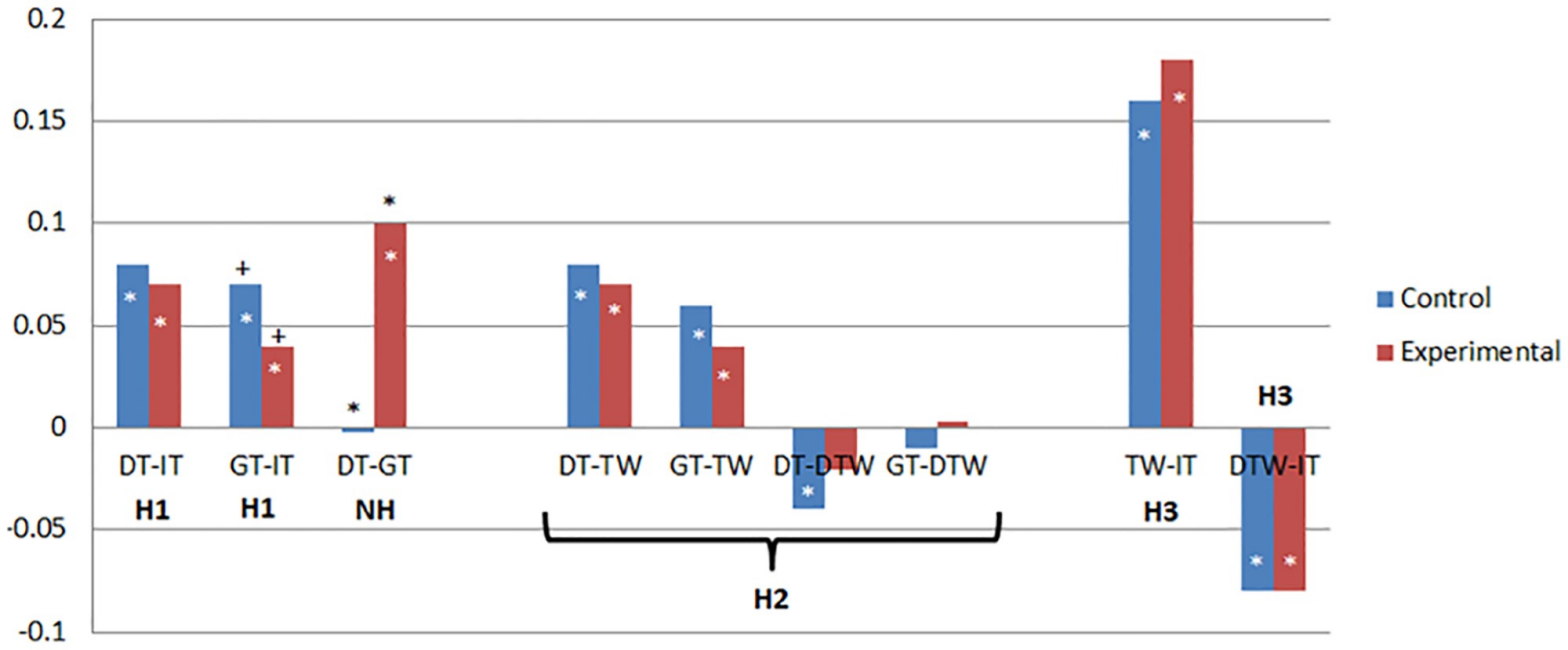
Covariances based on Multivariate Multilevel Model (MMLM) analyses. *Notes*. +p< .01, *p < .05; two-tailed, black outside bars = differences in covariances, white within bars = individual covariances. This figure is a graphical representation of the covariances listed in Tables 5, 6, 7 and 8. H1, H2, H3 indicate comparisons relevant to hypotheses 1, 2, and 3 respectively. H1 and H2 were that experimental would be < control. H3 was that experimental and control covariances would be different. NH indicates a comparison not hypothesized. DT = dispositional trust, GT = governmental trust, IT = institutional trust, TW = trustworthiness perceptions, DTW = distrustworthiness perceptions.

Having examined and modeled the effects of time, we next examined models relevant to our hypotheses. H1 stated that the relation between generalized trust and institutional trust over time would become smaller among those gaining knowledge (i.e., the experimental group), and thus would be greater for those who are and who remain less knowledgeable (i.e., the control group) compared to those who become more knowledgeable about the institution as time progressed (i.e., the experimental group). Although not a direct test of changes in relations over time, examining the within-participant (WP) residuals from the MMLM model is informative. Our results (reported in Table 5 and Fig 3) generally do not support H1. The WP covariance between dispositional trust and institutional trust (τ^2^_U11*U21_) was positive for both the control group (covariance = .08, *r* = .26, *p <* .01), and the experimental group (covariance = .07, *r* = .24, *p <* .01), and contrary to our hypothesis, there was no significant difference between the two groups (χ^2^(1) = 0.24, *p* = .62). Similarly, the WP covariance between governmental trust and institutional trust (τ^2^_U11*U31_) was not significantly greater for those in the control condition (covariance = .07, *r* = .28, *p <* .01) (S2 File, Note K) compared to those in the experimental condition (covariance = .04, *r* = .16, *p* < .01), although the difference was in the predicted direction, χ^2^(1) = 3.25, *p* = .07 (S2 File, Note L). Finally, although not hypothesized, there was a significantly greater governmental trust—dispositional trust covariance (τ^2^_U21*U31_, χ^2^(1) = 12.76, *p* < .001) among the experimental group (covariance = .10, *r* = .32, *p <* .01) than among the control group (covariance = -.002, *r* = -.01, *p =* .54).

**Table 5:** Multivariate multilevel model 1: Institutional trust, dispositional trust, and governmental trust.

Model Effects		Estimate	SE	*Z*-value	*p*-value
Cross-Variable Within-Participant Covariances					
Institutional Trust & Dispositional Trust Covariance, τ^2^_U11*U21_	Control	0.082***	0.022	1.182	< .001
	Experimental	0.070***	0.014	2.111	< .001
Institutional Trust & Governmental Trust Covariance, τ^2^_U11*U31_	Control	0.071***	0.017	4.118	< .001
	Experimental	0.036***	0.010	3.700	< .001
Governmental Trust & Dispositional Trust Covariance, τ^2^_U21*U31_	Control	-0.002 ^a^	0.023	0.500	0.309
	Experimental	0.096*** ^a^	0.014	1.815	< .001

*p < .05,

**p < .01,

***p < .001 significant parameter estimates

^a^ *p* < .001 differences between control group and experimental group estimates.

**Fig 4:**
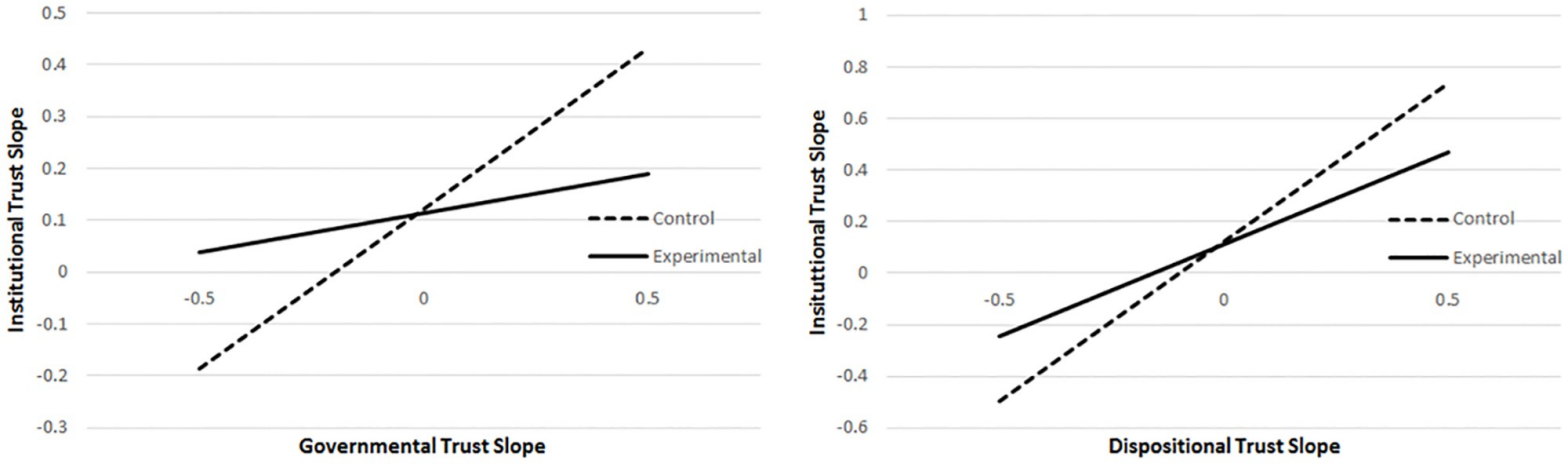
Institutional trust slope as predicted by governmental trust slope and dispositional trust slope for control and experimental groups. *Notes*. Predicted values based on slopes-as-outcomes models (see S1 Table) which indicated a significant difference between the experimental and control groups for the relationship between governmental trust and institutional trust slopes (left panel), but not for the relationship between dispositional trust and institutional trust slopes (right panel).

We also examined the “slopes-as-outcomes” model (see S1 Table for the full model) to explore changes in relationships over time. Our hypotheses were most concerned with changes over time (i.e., the model predicting the institutional trust slope, see Note M in S2 File) that differed between our control and experimental groups (i.e., involving interactions with manipulation). Our analyses indicated a significant interaction involving the institutional trust and governmental trust slopes (γ_19_ = -.47, *p* = .04), but not between the institutional trust and dispositional trust slopes (γ_18_ = -.52, *p* = .32). There also were no interactions involving governmental or dispositional trust WP residuals (γ_112_, γ_113_, *p*s > .20). The significant effect of governmental trust slope on institutional trust (γ_17_ = 0.62, *p* < .01) indicates, for control group participants, a greater change in institutional trust across surveys for those control participants with higher governmental trust slopes. The institutional trust slope × government trust slope × manipulation interaction (γ_19_ = -.47, *p* = .04) indicates there was a significant difference between manipulation groups for the effect of the governmental trust slope on the institutional trust slope (illustrated in Fig 4, left panel). While the effect of governmental trust slope on institutional trust slope was significant for those in the control group, it was not significant for those in the experimental group (experimental group *estimate* = .15, *p* = .31). Meanwhile, as illustrated in Fig 4 (right panel), the significant effect of dispositional trust slope on institutional trust slope indicates that participants in the control group with higher dispositional trust slopes showed a greater change in institutional trust across surveys (γ_16_ = 1.23, *p* < .01). Although the experimental group estimate appeared smaller (*estimate* = 0.71, *t*(720) = 1.93, *p* = .05), the non-significant interaction indicates it was not statistically different from the control group estimate.

In summary, the overall pattern of results was partly consistent with H1. We had expected the experimental group to begin with positive relations between generalized trust variables and institutional trust, and we expected this relation to become weaker over time. As hypothesized, there was some marginally significant (in the MMLM model) and statistically significant (in the slopes-as-outcomes model) evidence for this occurring for the governmental trust variable, but these effects only involved relations between changes over time (slopes) and not relations between WP residuals. Contrary to our hypothesis, a similar pattern involving dispositional trust did not achieve statistical significance.

#### 3.3.2. Hypothesis 2: Generalized trust predicting perceptions of trustworthiness

Our second hypothesis (H2) postulated that, among the experimental group only, the relationships between generalized trust variables and trustworthiness variables (including positive trustworthiness and distrustworthiness) also should decrease over time. To avoid problems with high collinearity, we examined trustworthiness and distrustworthiness in separate models. Again, examination of the within-participant (WP) residuals from the MMLM results is informative. The first model (Table 6, see also Fig 3, H2 comparisons) is similar to the model used to test H1, but included trustworthiness in the model instead of institutional trust. As expected, the WP covariance between dispositional trust and trustworthiness (τ^2^_U11*U21_) was significant in the positive direction for both those in the experimental group (covariance = .07, *r* = .27, *p <* .01) and control group (covariance = .08, *r* = .28, *p <* .01). However, contrary to our hypothesis, the covariances did not significantly differ (χ^2^ (1) = 0.17, *p* = .68), as would be expected if the experimental group covariance decreased over time. Similarly, the WP covariance between governmental trust and trustworthiness was positive for both experimental and control groups (covariances = .04, .06, *r*s = .21, .26, *p*s *<* .01, respectively), and it did not significantly differ between groups (χ^2^ (1) = 0.12, *p* = .28).

**Table 6:** Results from multivariate multilevel model 2: Trustworthiness, dispositional trust, and governmental trust.

Model Effects		Estimate	SE	*Z*-value	*p*-value
Cross-Variable Within-Participant Covariances					
Trustworthiness & Dispositional Trust Covariance, τ^2^_U11*U21_	Control	0.077***	0.019	4.063	< .001
	Experimental	0.068***	0.012	5.667	< .001
Trustworthiness & Governmental Trust Covariance, τ^2^_U11*U31_	Control	0.056***	0.015	3.733	< .001
	Experimental	0.038***	0.009	4.222	< .001
Governmental Trust & Dispositional Trust Covariance, τ^2^_U21*U31_	Control	-0.002 ^a^	0.023	0.087	.465
	Experimental	0.096***^a^	0.014	6.857	< .001

*p < .05,

**p < .01,

***p < .001 significant parameter estimates

^a^ Indicates *p* < .001 differences between control group and experimental group estimates (this difference is the same as in Table 5 because governmental and dispositional trust were in both Table 5 and Table 6 models).

**Fig 5:**
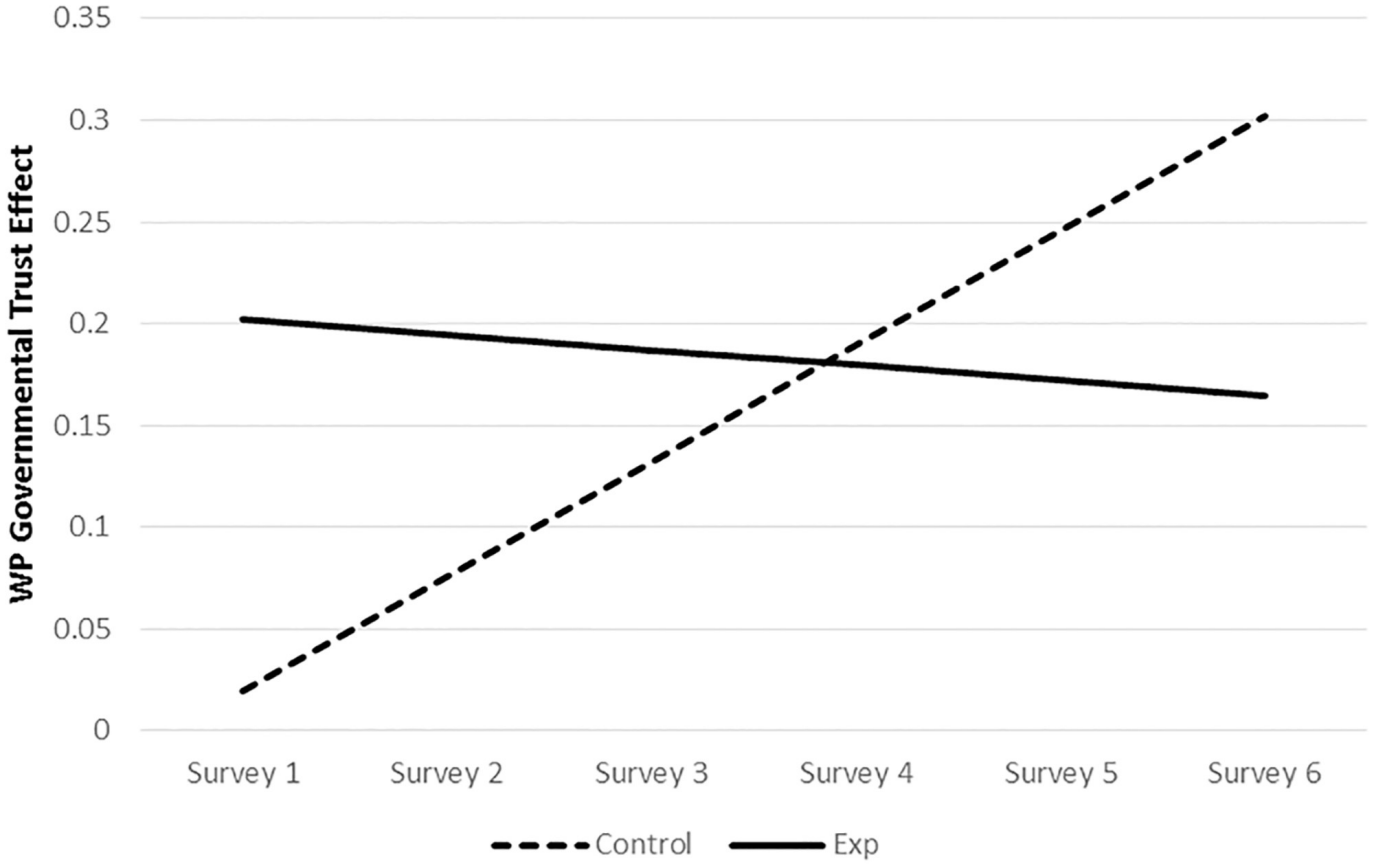
Within-Person (WP) deviation from predicted governmental trust ratings effect on trustworthiness slope over time, for control and experimental groups. *Note*. Figure illustrates the marginal three way interaction between WP governmental trust, time, and experimental manipulation prediting change in trustworthiness perceptions over time (i.e., trustworthiness slope) as described in note 15 (see also S2 Table).

Examination of the slopes-as-outcomes model (see S2 Table for full model) indicates the generalized trust and positive trustworthiness slopes were positively related, as expected. For the control group, a 1-point increase in dispositional trust slope corresponded to a trustworthiness slope significantly increasing by 0.89 (γ_16_, *p* < .01). Meanwhile, a 1-point increase in the governmental trust slope predicted a significant increase of 0.41 in the trustworthiness slope (γ_17_, *p* < .01). The lack of significant interactions indicated that the experimental and control groups did not differ on these effects. In addition, the WP dispositional trust effect did not significantly relate to trustworthiness and did not significantly differ between manipulation groups (S2 Table, γ_110_, γ_112_). Finally, the 3-way interaction between WP governmental trust, time, and manipulation was in the correct direction (specifically, indicating more decrease over time of the covariance for the experimental group than the control group, which instead increased, as illustrated in Fig 5), but only marginally significant (γ_113_ = -0.06, *t*(255) = 1.71, *p* = .09) (S2 File, Note N).

Next, we examined the pattern of findings replacing trustworthiness with distrustworthiness in our models (see Table 7, see also Fig 3, H2 comparisons). As expected, the within-participant covariance between dispositional trust and distrustworthiness (τ^2^_U11*U21_) was negative for both those in the control group (covariance = -.04, *r* = -.13, *p =* .03) and the experimental group (covariance = -.02, *r* = -.05, *p =* .13), although the relationship was not significant for the experimental group. Contrary to our hypotheses, however, this difference was not significantly different between groups, (χ^2^(1) = 0.92, *p* = .34). Also contrary to our hypotheses, the within-participant correlation between governmental trust and distrustworthiness was not significant for either group, and the groups did not significantly differ (χ^2^(1) = 0.48, *p* = .49).

**Table 7:** Multivariate multilevel model 3: Distrustworthiness, dispositional trust, and governmental trust.

Model Effects		Estimate	SE	*Z*-value	*p*-value
Between-Variable Within-Participant Covariances					
Distrustworthiness & Dispositional Trust Covariance, τ^2^_U11*U21_	Control	-0.041*	0.021	1.905	0.028
	Experimental	-0.016	0.014	1.143	0.127
Distrustworthiness & Governmental Trust Covariance, τ^2^_U11*U31_	Control	-0.011	0.016	0.688	0.754
	Experimental	0.003	0.010	0.300	0.382
Governmental Trust & Dispositional Trust Covariance, τ^2^_U21*U31_	Control	-0.002^a^	0.023	0.087	0.465
	Experimental	0.096*** ^a^	0.014	6.857	< .001

*p < .05,

**p < .01,

***p < .001 significant parameter estimates

^a^ Indicates *p* < .05 differences between control group and experimental group estimates.

The slopes-as-outcomes model also produced little evidence of any differences between groups (see S3 Table) when predicting distrustworthiness. Again, our hypotheses were most concerned with interactions involving both time (i.e., the model for the slopes) and manipulation (i.e., interactions involving manipulation effects). However, none of the interactions involving manipulation were significant (*p*s ≥ .11).

In summary, we found only slight evidence in support of H2 when examining positive trustworthiness. As reported above, only one of four interactions approached significance. None of the interactions involving distrustworthiness were significant or marginally significant.

#### 3.3.3. Hypothesis 3: Perceptions of trustworthiness predicting institutional trust

Our third hypothesis (H3) proposed that as participants become more knowledgeable about the water regulatory institutions across time (i.e., those in the experimental condition), we expected changes in the relative ability of specific trustworthiness judgments to predict institutional trust. For example, certain facets of trustworthiness (e.g., our positive trustworthiness or distrustworthiness scales) may remain closely related to institutional trust, while others become less closely related. Because of the high collinearity of our variables (see Table 3), we conducted two separate MMLM models predicting institutional trust, one including positive trustworthiness as the predictor, and one including distrustworthiness. Once again, examination of the averages is useful. As can be seen in Table 8 (see also Fig 3, H3 comparisons), our results did not support our hypothesis. The experimental and control group average within-participant covariances did not differ significantly when examining either the covariances between institutional trust and trustworthiness (*τ*^*2*^_U11*U21_, experimental and control respective covariances = .18, .16, *r* = .87, .87, χ^2^ (1) = 0.38, *p* = .54) or between institutional trust and distrustworthiness (*τ*^*2*^_U11*U21_ = -.08, -.08, *r* = -.24, -.22, χ^2^(1) = 0.00, *p* = .95).

**Table 8:** Multivariate multilevel models 3 and 4: Institutional trust and positive trustworthiness, and institutional trust and distrustworthiness.

Model Effects		Estimate	SE	*Z-*value	*p*-value
Cross-Variable Within-Participant Covariances					
Institutional Trust & Trustworthiness Covariance, τ^2^_U11*U21_	Control	0.175***	0.017	10.29	< .001
	Experimental	0.162***	0.011	14.73	< .001
Institutional Trust & Distrustworthiness Covariance, τ^2^_U11*U31_	Control	-0.078***	0.016	4.88	< .001
	Experimental	-0.077***	0.011	7.00	< .001

***p < .001 significant parameter estimates

**Fig 6:**
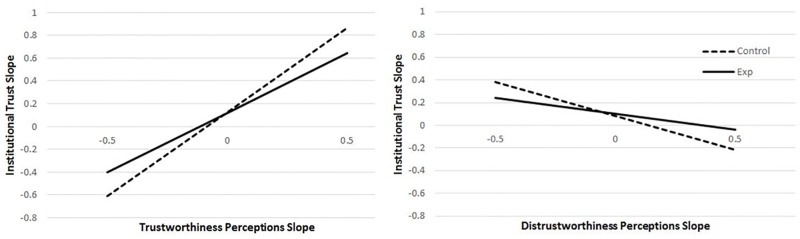
Effects of trustworthiness and distrustworthiness perceptions slopes on institutional trust slope for control and experimental groups. *Notes*. Predicted institutional trust slopes derived from slope-as-outcomes models (see full results in S4 and S5 Tables).

Examination of the “slopes-as-outcomes” model predicting institutional trust from trustworthiness and time (see S4 Table) indicated a significant difference between groups in the effects of trustworthiness slope on institutional trust slope (γ_15_ = -0.43, *p* < .01). As shown in Fig 6 (left panel), for every 1-point increase in a control participant’s trustworthiness slope, the institutional trust slope significantly increased by 1.48 (γ_14_, *p* < .01). However, that effect was significantly lower for those in the experimental group (*estimated effect* = 1.05, *p* < .01). Thus, the effect of overall change in trustworthiness perceptions over time on overall change in institutional trust was stronger for the control group and weaker (though still significant) for the experimental group. This is consistent with our hypothesis that experimental group members would form distinctions among our three types of trust variables (generalized trust variables, trustworthiness perceptions, and expressions of direct institutional trust). However, the pattern of results was different for the model including distrustworthiness as the predictor (full results shown in S5 Table, see also Fig 6, right panel). In this model, there were no significant differences between the experimental and control groups in the relations between institutional trust and distrustworthiness slopes over time.

#### 3.3.4. Hypothesis 4: Changes in the relations between Within-Person (WP) trustworthiness perceptions and institutional trust over time

**Fig 7:**
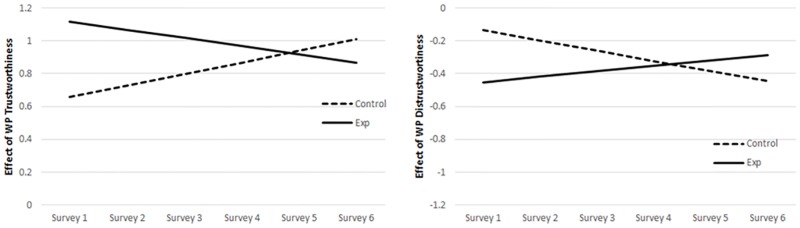
The effects of Within-Person (WP) trustworthiness perceptions (top panel) and distrustworthiness perceptions (lower panel) on institutional trust over time, for the experimental and control groups. *Notes*. Effects estimated based on slopes-as-outcomes models as described in the texts. Although patterns of differences in change over time were consistent with our hypotheses, only the difference in effects of WP Trustworthiness achieved statistical significance.

H4 stated that among those in the experimental condition, institutional trust would become less associated with within-participant fluctuations in various trustworthiness perceptions (including trustworthiness and distrustworthiness). Examination of the “slopes-as-outcomes” models used above for H3 (full models reported in S4 and S5 Tables) provides information relevant to these hypotheses. Results indicated a significant 3-way interaction between WP trustworthiness, time, and manipulation (S4 Table, γ_17 =_ -.12, *p* < .01) predicting institutional trust. The pattern of this interaction (illustrated in Fig 7) was such that the WP effect of trustworthiness at survey 1 (i.e., time 0) was significantly positive for the control group (γ_06_ = .66, *p* < .01) and the experimental group (*estimate* = 1.12, *p* < .01), with the experimental group having a significantly higher effect (γ_07_ = .46, p < .01). The control group’s relations between WP trustworthiness and one’s institutional trust increased as the study progressed (γ_16_ = .07, *p* = .04). However, for the experimental group the effect of WP trustworthiness (i.e., deviation from one’s predicted rating at any given survey) on one’s institutional trust became less positive over time (*estimate* = -.05, *t*(590) = 2.24, *p* = .03), with a final effect of .87 at Survey 6 (*t*(318) = 11.05, *p* < .01). This is consistent with our hypothesis (H4), but support was found only for trustworthiness, and not for distrustworthiness. For distrustworthiness, results indicated the 3-way interaction between WP distrustworthiness, time, and manipulation was not significant (S5, γ_17 =_ .10, *p* = .12).

## 4. Discussion

Previous research and theory have proposed that the relationship between institutional trust and generalized trust depends upon the individual’s knowledge of the institution [18, 57, 58]. This study tested the effect of knowledge gains on institutional trust over time. Our manipulation checks confirmed that participants in the experimental condition experienced statistically significant greater increases in subjective knowledge than those in the control condition, but the difference in objective knowledge increase between the two conditions was not significant. Our data therefore only speak to how trust changes as subjective knowledge increases. This is an important qualification given that prior research finds only a modest relation between subjective and objective knowledge [86, 87].

In addition, both the experimental and control group showed increases in institutional trust that were stronger at the beginning of the study and plateaued over time. Because the control group was not exposed to the additional information about water regulatory institutions, this pattern may be more a result of repeated measurement and diffuse familiarity over time than increases in knowledge, per se.

### 4.1. Impacts of generalized trust variables

Our first hypothesis (H1) was that the generalized trust constructs—such as propensity to trust people or government in general—would become less important as a basis for trust in a specific institution as participants in the experimental condition gained in knowledge of the institution. Our findings partially supported this hypothesis, suggesting that those receiving information about water-regulatory institutions discriminated between governmental trust and trust in water institutions more than the control group.

Because our knowledge-centered theory of trust posits that trustworthiness, institutional trust, and generalized trust constructs start out in an undifferentiated form, with trustworthiness perceptions and institutional trust both being initially estimated from generalized trust, our second hypothesis (H2) took the same form as H1, but it substituted trustworthiness (and distrustworthiness) variables for institutional trust. However, save for one marginal effect, none of our results supported H2. The only support for H2 came from a marginal interaction effect found in our slopes-as-outcomes models, involving governmental trust becoming less predictive of change in institutional trustworthiness in the case of the experimental group, but more predictive in the case of the control group.

The fact that the hypothesized (H1 and H2) patterns were not supported by analyses involving dispositional trust as the generalized trust variable merits additional discussion. Our data consistently supported the conclusion that *dispositional trust* (as opposed to governmental trust) was *not* a significantly weaker predictor of either institutional trust or perceptions of trustworthiness (and distrustworthiness) among those increasing in institutional knowledge. This undermines our theorized role of generalized trust. Specifically, our study finds that dispositional trust may play a more active and ongoing role, continuing to affect institutional trust even as an individual’s knowledge increases. This is more consistent with the suggestion that generalized trust’s influence may actually increase as relationships “thicken” and as these relationships feature greater interpersonal contact [23]. Although interpersonal contact did not increase here, increased sophistication could, in some cases, humanize institutions in a way that makes generalized trust more relevant over time instead of less. This is also consistent with some personality research showing that certain individual differences may increase the coherence of certain traits and outcomes over time [88, 89]. Our experiment was intended to resemble how everyday people might learn about institutions in their everyday lives—that is, through non-intense media exposure. In such contexts, it appears that generalized trust variables (especially dispositional trust) may continue to exert substantial effects on institutional trust and its development over repeated information exposures.

### 4.2. Other bases for institutional trust

Examination of our data also indicated partial but inconsistent support for our prediction that experimental participants would begin to base their trust in the institution on different trustworthiness (and distrustworthiness) factors than the control group. Our prediction was based on our hypothesis (H3) that the relationships between institution-specific trustworthiness variables and trust in a target institution would alter with experiences with that institution. It appears that receiving regular information about the institutions significantly attenuated the association between perceived trustworthiness and trust. But there were no significant differences between the experimental and control groups in the extent to which change in perceptions of distrustworthiness predicted change in institutional trust. Consistent with perspectives that view trust and distrust as separate constructs rather than opposite ends of the same continuum [90, 91], this may suggest that different models are needed to represent how perceptions of distrustworthiness predict unspecified institutional trust as people gain knowledge of an institution over time. Consistent with the idea that “bad is stronger than good” [92], it may be that distrustworthiness perceptions continue to be more powerful predictors, whereas trustworthiness perceptions more quickly lose their power.

We also predicted that exposure to information about the water regulatory institutions would result in the development of more stable levels of institutional trust, which are less influenced by momentary fluctuations (from one’s developmental trajectory) in trustworthiness/distrustworthiness perceptions (H4). When examining trustworthiness (but not distrustworthiness), this seemed to be the case for our experimental group. As hypothesized, the effect of within-person trustworthiness perceptions on institutional trust became significantly less positive over time for the experimental group. However, for the control group, the significant predictive effect of trustworthiness fluctuations from one’s developmental trajectory on one’s institutional trust grew stronger over time rather than decreasing. Although we had not predicted such an increase in the control group (we had hypothesized that the control group would not show change in this regard), this effect may be a result of repeated exposure to the measures pertaining to the water regulatory institutions, which may have sensitized participants to information about the institutions and increased the coherence of their responding. Likewise, the fact that the control group significantly increased in its institutional trust (as did the experimental group) may be, in part, because repeated exposure to questions about the water regulatory institutions increased familiarity, which tends to increase positive evaluations of a target [93, 94].

### 4.3. Strengths, limitations, and future directions

Our main objective in this research was to investigate a knowledge-centered theory of trust which posits relationships among generalized trust, trustworthiness constructs, and institutional trust, with specific consideration of the extent to which institutional knowledge moderates those relationships over time. Thus, our findings contribute to the trust literature in organizational behavior, social psychology, political science, and, because of our use of water regulatory institutions specifically, natural resource management. Little prior research on trust has taken a longitudinal approach. Although developmental approaches to trust models have been proposed [95], prior research has not examined the role that institutional knowledge plays in the evolution of institutional trust and in potentially altering the reasons for (bases of) that trust. Our research begins to address this gap, empirically and theoretically, contributing to a more integrated view of how institutional trust evolves over time.

One substantial advantage of our study over prior studies is that participants were randomly assigned to conditions in which they regularly read naturalistic information, about either water regulatory institutions or an unrelated institution, over a relatively long (i.e., 15-month) time-period. Participants in both conditions began the study with very little knowledge of water regulatory institutions and, because only the experimental group regularly read about water regulatory institutions, our design allowed us to distinguish changes due to time and repeated measures from those due to our experimental manipulation. A second advantage is our focus on understanding the factors predicting “unspecified” measures of institutional trust commonly used by large-scale surveys to monitor public trust in specific institutions. This focus makes our research highly relevant to theoretical issues raised in that very large literature, as well as to efforts to reform public policy.

Limitations of our study point to the need and opportunity for further research. Our measures of subjective and objective knowledge suggested that our two groups differed significantly in subjective but not in objective knowledge. Given the different effects of these two types of knowledge found in other research, it is possible that greater support for our emerging theory may have been found if objective knowledge had also differed significantly between groups. Also, our measures of unspecified trust versus perceptions of trustworthiness and distrustworthiness were reliable but low in discriminant validity. Future research is needed to better understand the situations or contexts in which trustworthiness and distrustworthiness perceptions are more versus less able to be distinguished from institutional trust [16].

Further study of how commitment to an institution relates to trust would be a fruitful future direction. In particular, researchers might focus on how the degree to which a sense of loyalty or felt obligation to an institution [63] is stable and persistent within an individual [15, 60–62]–perhaps more so than trustworthiness perceptions of an institution’s benevolence, competence, and so on. For example, to what extent are loyalty and a perceived obligation to obey an institution shaped more by extended relations with an institution than by media exposure and immediate interactions [66, 67]? As we briefly mentioned earlier, commitment has been examined as both a basis and outcome of trust [15, 19, 68]. Although it seems unlikely that trustors would *specifically* feel much loyalty or obligation to an unknown or unfamiliar institution, as knowledge is gained institutional trust (e.g., based on trustworthiness perceptions) may facilitate the development of loyalty or perceived obligation or even crystalize a strong and stable rejection of loyalty or obligation [68]. These commitments in particular may subsequently be bases for institutional trust (and distrust) in the future [69].

Future directions might also include other ways of operationalizing our trust variables. For instance, rather than using scales aimed at measuring individuals’ generalized trust, perhaps participants’ propensities to trust might be measured according to their behavior during trust games such as those widely used in behavioral economics research [96, 97]. Further, future research should be conducted with participants who are not college students given concerns about the representativeness of college students’ attitudes [98].

### 4.4. Conclusions

This study provided a rigorous, experimental test of how institutional trust and its bases change or stay the same as people gain information about and increase in subjective knowledge about an institutional target over time. The reliable finding that dispositional trust continues to predict institutional trust and perceptions of trustworthiness over time strongly suggests that its influence is important in many situations where people are learning more about institutions through media exposure. We found little support for the idea that (subjectively) “knowing more” leads to less reliance upon dispositions. Institutions interested in increasing public trust should not assume that providing more and more information will overcome such dispositional influences. However, our evidence that governmental trust may begin to de-couple from institutional trust over time does suggest that people form more nuanced distinctions that allow them to distinguish among institutions and their trust for each one. To the extent that an institution’s public trust might benefit from being distinguished from trust in other institutions, provision of additional information could assist with that goal.

## Supporting information

S1 File Appendix of measures. (DOCX)

S2 File Notes. (DOCX)

S3 File Longitudinal data. (CSV)

S4 File Formatting. (SAS)

S5 File Longitudinal syntax. (SAS)

S1 Table Slopes-as-outcomes model 1: Dispositional and governmental trust predicting institutional trust. (DOCX)

S2 Table Slopes-as-outcomes model 2: Dispositional and governmental trust predicting trustworthiness. (DOCX)

S3 Table Slopes-as-outcomes model 3: Dispositional and governmental trust predicting distrustworthiness. (DOCX)

S4 Table Slopes-as-outcomes model 4: Trustworthiness predicting institutional trust. (DOCX)

S5 Table Slopes-as-outcomes model 4: Distrustworthiness predicting institutional trust. (DOCX)
