# Supplementary material for: A longitudinal and experimental study of the impact of knowledge on the bases of institutional trust
Source: PLoS One. 2017 Apr 17;12(4):e0175387. doi: 10.1371/journal.pone.0175387 (PMC5393579; doi:10.1371/journal.pone.0175387)
Supplement: S1 File — (DOCX) [file pone.0175387.s001.docx]

## Appendix of Measures

### Unspecified Institutional Confidence

1. My confidence in the [institution] is high
2. The [institution] does its job well
3. I have confidence in the [institution] to do its job
4. I believe the [institution] will perform its functions as it should

### Dispositional Trust

1. Generally speaking, I would say that most people can be trusted.
2. I believe that people are basically moral.
3. I believe in human goodness.
4. I believe most people try to be fair.
5. I trust others.
6. I think that most people would try to be fair.
7. I would say that most of the time people try to be helpful.
8. I believe that others have good intentions.
9. I trust what people say.

### Governmental Trust

1. I can trust the federal government in Washington, D.C., to do their job well.
2. I can trust the state government to do their job well.
3. I can trust the local government to do their job well.
4. I can trust the administration at the University of Nebraska to do their job well.
5. I can trust the United States Supreme Court to do their job well.
6. I can trust the United States military to do their job well.
7. I can trust the President to do his job well.

### Trustworthiness

**Legitimacy**

1. The decision makers of the [institution] are selected using fair procedures
2. The procedures followed by the [institution] are lawful
3. The [institution] uses its power appropriately
4. The [institution] is a legitimate authority on water regulation

**Respect**

1. Most members of the [institution] treat people with respect
2. I think the [institution] respects my rights
3. Even when dealing with people who disagree with it, the [institution] still treats people with dignity

**Voice**

1. I feel like the [institution] listens to the opinions of the people it regulates
2. Residents have great say in important [institution] decisions
3. Citizens can influence the [institution] decisions

**Distributive Justice**

1. Water resources are allocated fairly by the [institution]
2. The amount of water I receive is fair
3. My allocation of water is fair compared to what others receive

**Loyalty**

1. The [institution] has the right amount of power
2. I am steadfast in my support of the [institution]
3. I would support the [institution] even if I disagree with some of its specific decisions or policies
4. Even when I disagree with a decision made by the [institution], I still believe it deserves respect

**Values Similarity**

1. I believe the [institution] shares my values about how water should be regulated
2. To the extent that I understand them, I share the [institution] values about how water should be regulated.
3. I believe that the [institution] supports my values about water allocation.

**Benevolence**

1. For the most part, the decisions made by the [institution] are made out of care and concern for area residents
2. Most decision makers of the [institution] care about residents in the area they regulate
3. The decision makers of the [institution] put aside their own personal interests in making decisions that are right for the community.

**Competence**

1. Most decision makers of the [institution] are competent to do their jobs
2. Most decision makers of the [institution] are highly qualified individuals

**Integrity**

1. The [institution] is honest
2. Even when it is difficult, the [institution] still maintains its values.
3. Most [institution] decision makers have integrity

**Obligation to Obey**

1. I feel I should accept decisions made by the [institution]
2. Good citizens will obey water regulations set for an area by the [institution]
3. People should follow the decisions and policies of the [institution] even when they disagree with them
4. Even in the face of personal difficulty, I would not violate the regulations of the [institution]

### Untrustworthiness

**Bias**

1. I think the [institution] acts in the interests of some groups over others
2. The decisions made by the [institution] are biased
3. The [institution] is overly influenced by special interest groups
4. The [institution] can be trusted to make decisions that are right for the entire community

**Cynicism**

1. The [institution] does not protect my interests.
2. The [institution]is not representative of Nebraskans
3. The [institution] is out of touch with what’s going on in its communities
4. The decision makers of the [institution] are primarily motivated to do whatever they need to stay in power
