# Supplementary material for: A longitudinal and experimental study of the impact of knowledge on the bases of institutional trust
Source: PLoS One. 2017 Apr 17;12(4):e0175387. doi: 10.1371/journal.pone.0175387 (PMC5393579; doi:10.1371/journal.pone.0175387)
Supplement: S2 File — (DOCX) [file pone.0175387.s002.docx]

# Notes

A Theorists sometimes distinguish trust and confidence—for example, on the bases of whether it is consciously considered [1, 2], whether it stems from expectations of shared values versus assessments of past performance [3], or based on the interpersonal versus institutional target of trust [4]. We do not make this distinction, in part due to these inconsistencies in the distinctions, as described in more detail elsewhere [5]. In addition, respondents treat the wording “I have confidence in [institution]” and “I trust [institution]” as approximately equivalent [6]. Therefore, we treat the constructs as synonymous here.

B For example, see the trust in government data provided by the Pew Research Center at <http://www.people-press.org/2014/11/13/public-trust-in-government/>, or by the General Social Survey at <https://gssdataexplorer.norc.org/>.

C We do not believe that the progression from relying on internal dispositions to specific institutional assessments to committed bases will form three completely distinguishable discrete stages, or that the development of trust over time necessarily involves stage-like “jumps” as opposed to a more gradual transition. Rather, we invoke stages as a heuristic. The more important point is that knowledge gains will affect institutional trust judgments by changing their bases.

D A total of 101 participants completed all six surveys, 17 completed five surveys, 19 completed four surveys, 31 completed three surveys, and 17 completed two surveys. We conducted one-way ANOVAs with the total number of surveys completed as the independent variable and found no significant differences between groups on our dependent variables, assignment to manipulation group, age, or gender.

E We conducted all analyses with a variable to test for main and interactive effects of type of science course (environmental vs. other science) from which students were recruited, and found no effects. Therefore, this potential influencing factor is not discussed further.

F The specific 12 items used at each survey varied somewhat because we replaced some poorer performing/less precisely worded items from earlier surveys with new items at later surveys. Although this makes it difficult to compare changes in objective knowledge over time, we were most interested in the relative knowledge of participants in different groups (e.g., over time did the students reading about water regulatory agencies get more answers correct than those control students who read about health and human services?). Because participants in each (control and experimental) group always answered the same knowledge questions as other participants at a given time point (survey), we were able to make these comparisons.

G Current and prior examinations of these items and measures indicated the items represented the targeted trustworthiness constructs as intended, but the construct measures were often highly correlated [6]. We examined the potential of reducing the scales to a smaller number, using principal components analysis (PCA), entering each of the trustworthiness scales (i.e., cynicism, respect, etc.) from the first survey as variables, and using oblique (Promax) rotation with a Kappa value of 4, as recommended by Hendrickson and White (7). Examination of the PCA suggested that our trustworthiness scales formed two factors (*r* = -.61) with eigenvalues for each factor, ignoring the other factor, of 8.40 and 4.79, respectively. The first factor, labeled *positive trustworthiness*, contained all 10 scales related to positive perceptions of institutions (i.e., legitimacy, respect, loyalty, obligation to obey, voice, distributive justice, shared values, benevolence, competence, integrity). Each scale in the trustworthiness factor loaded positively and strongly, with a minimum factor loading of .81 (benevolence) and a maximum factor loading of .95 (loyalty). The second factor, labeled *distrustworthiness*, contained the two scales related to negative perceptions of institutions, with both loading positively and strongly (bias = .90, cynicism = .97). These results are consistent with prior findings from similar investigations of the factor structure of these constructs [6].

H Separate PCAs of the governmental and dispositional trust items suggested that each formed a single factor (eigenvalues of 3.89 and 5.01, respectively). The full set of governmental trust items included questions regarding the Department of Natural Resources and Natural Resource Districts, but these questions were not included in the generalized governmental trust scale.

I There was not a significant relation between manipulation and survey 1 (time 0) intercepts of any of our variables, further supporting that random assignment to manipulation was successful.

J Because the parameter estimates from these models and the models including manipulation are so similar, we report both sets of results in text, but present a table of parameter estimates only from the models that are conditional upon manipulation (see Table 4).

K The tables report covariances. For easier interpretation, in text we also report correlations, with covariance to correlation conversions conducted using the formula: *r_xy_* = *cov_xy_* / SQRT (*var_x_ * var_y_*)

L That is, for any completed survey where a participant reported high governmental trust (relative to his or her predicted value), he or she was marginally more likely to also report high institutional trust (relative to his or her predicted value) if he or she was in the control group compared to the experimental group.

M Although there were several other significant effects influencing the institutional trust intercept, these effects do not directly address our hypotheses. Therefore, we did not report these results in detail (see supplemental S1 Table).

N Because it did approach significance, it may be worthwhile to examine the pattern of this marginal interaction to see if it was in the hypothesized direction or not. The pattern was that, for the control group, at survey 1 (time 0), within-person deviation from predicted governmental trust ratings did not significantly predict one’s trustworthiness slope (effect = 0.02, *p* = .81). However, that effect became positive over time, such that at survey 6 (time 5) the estimated effect was significantly positive (effect = 0.30, *p* < .01). In contrast, for the experimental group, at survey 1, WP deviation from predicted governmental trust ratings significantly predicted more positive trustworthiness slopes (effect = 0.20, *p* < .01). While that effect decreased over time, it remained significantly positive (time 5 effect = 0.17, *p* = .03). The pattern for the experimental group is consistent with our predictions. The pattern for the control group was not predicted by our theoretical model but is consistent with a similar finding (see H4 analyses) indicating that WP variance in trustworthiness perceptions became increasingly predictive of change in institutional trust over time.

**References Cited in Notes**

1. Giddens A. The consequences of modernity. Cambridge: Cambridge University Press; 1996.

2. Luhmann N. Familiarity, confidence, trust: Problems and alternatives. In: Gambetta D, editor. Trust: Making and breaking cooperative relations. Oxford: Basil Blackwell; 1988. p. 94-107.

3. Earle TC, Siegrist M. Morality information, performance information, and the distinction between trust and confidence. Journal of Applied Social Psychology. 2006;36(2):383-416.

4. Cao L. Differentiating confidence in the police, trust in the police, and satisfaction with the police. Policing: An International Journal of Police Strategies & Management. 2015;38(2).

5. PytlikZillig LM, Kimbrough CD. Consensus on conceptualizations and definitions of trust: Are we there yet? In: Shockley E, Neal TM, PytlikZillig LM, Bornstein BH, editors. Interdisciplinary perspectives on trust: Towards theoretical and methodological integration. New York: Springer; 2016. p. 17-47.

6. PytlikZillig LM, Hamm JA, Shockley E, Herian MN, Neal TM, Kimbrough CD, et al. The dimensionality of trust-relevant constructs in four institutional domains: Results from confirmatory factor analyses. Journal of Trust Research. 2016. doi: <http://dx.doi.org/10.1080/21515581.2016.1151359>.

7. Hendrickson AE, White PO. Promax: A quick method for rotation to oblique simple structure. British Journal of Statistical Psychology. 1964;17(1):65-70.
