# Supplementary material for: A longitudinal and experimental study of the impact of knowledge on the bases of institutional trust
Source: PLoS One. 2017 Apr 17;12(4):e0175387. doi: 10.1371/journal.pone.0175387 (PMC5393579; doi:10.1371/journal.pone.0175387)
Supplement: S1 Table — (DOCX) [file pone.0175387.s006.docx]

S1 Table

*Slopes-as-Outcomes Model 1: Dispositional and Governmental Trust Predicting Institutional Trust.*

| Model Effects | Estimate | SE | *DF* | *t*-value | *p*-value |
| --- | --- | --- | --- | --- | --- |
| Model for the Means  Institutional Trust Intercept (Survey 1 Ratings), β_0_ |  |  |  |  |  |
| γ_00_ Intercept | 4.941 | 0.088 | 93 | N/A | N/A |
| γ_01_ Manipulation Effect (0 = Control, 1 = Experimental) | 0.101 | 0.099 | 143 | 1.02 | .307 |
| γ_02_ Dispositional Trust Intercept (0 = mean, 5.436) | 0.489** | 0.167 | 84 | 2.93 | .004 |
| γ_03_ Governmental Trust Intercept (0 = mean, 5.031) | 0.293** | 0.104 | 86.1 | 0.39 | .006 |
| γ_04_ Dispositional Trust Intercept × Manipulation Effect | -0.257 | 0.202 | 164 | 1.27 | .205 |
| γ_05_ Governmental Trust Intercept × Manipulation Effect | 0.096 | 0.36 | 211 | 0.70 | .484 |
| γ_06_ Dispositional Trust Slope | 1.209 | 1.961 | 89 | 0.62 | .539 |
| γ_07_ Governmental Trust Slope | -2.249* | 0.893 | 89.3 | 2.52 | .014 |
| γ_08_ Dispositional Trust Slope × Manipulation Effect | -4.280 | 2.563 | 215 | 1.67 | .097 |
| γ_09_ Governmental Trust Slope × Manipulation Effect | 2.721* | 1.116 | 192 | 2.44 | .016 |
| γ_010_ Dispositional Trust Residual (WP Effect) | 0.325* | 0.138 | 707 | 2.36 | .019 |
| γ_011_ Governmental Trust Residual (WP Effect) | -0.011 | 0.093 | 706 | 0.12 | .905 |
| γ_012_ Dispositional Trust Residual × Manipulation Effect | -0.213 | 0.167 | 707 | 1.28 | .203 |
| γ_013_ Governmental Trust Residual × Manipulation Effect | 0.178 | 0.114 | 706 | 1.56 | .119 |
|  |  |  |  |  |  |
| Linear Time Slope (0 = Survey 1), β_1_ |  |  |  |  |  |
| γ_10_ Intercept | 0.122*** | 0.034 | 717 | 3.59 | < .001 |
| γ_11_ Manipulation Effect | -0.009 | 0.020 | 737 | 0.45 | .653 |
| γ_12_ Dispositional Trust Intercept | -0.016 | 0.032 | 728 | 0.47 | .636 |
| γ_13_ Governmental Trust Intercept | 0.022 | 0.020 | 724 | 1.10 | .271 |
| γ_14_ Dispositional Trust Intercept × Manipulation Effect | 0.024 | 0.041 | 728 | 0.58 | .562 |
| γ_15_ Governmental Trust Intercept × Manipulation Effect | 0.015 | 0.028 | 727 | 0.53 | .598 |
| γ_16_ Dispositional Trust Slope | 1.233*** | 0.370 | 711 | 3.33 | < .001 |
| γ_17_ Governmental Trust Slope | 0.619*** | 0.169 | 718 | 3.65 | < .001 |
| γ_18_ Dispositional Trust Slope × Manipulation Effect | -0.520 | 0.523 | 716 | 0.99 | .320 |
| γ_19_ Governmental Trust Slope × Manipulation Effect | -0.468* | 0.225 | 720 | 2.08 | .038 |
| γ_110_ Dispositional Trust Residual (WP Effect) | -0.043 | 0.039 | 707 | 1.09 | .277 |
| γ_111_ Governmental Trust Residual (WP Effect) | 0.049 | 0.036 | 709 | 1.37 | .173 |
| γ_112_ Dispositional Trust Residual × Manipulation Effect | 0.020 | 0.052 | 709 | 0.39 | .699 |
| γ_113_ Governmental Trust Residual × Manipulation Effect | -0.056 | 0.044 | 709 | 1.27 | .204 |
|  |  |  |  |  |  |
| Quadratic Time Slope, β_2_ |  |  |  |  |  |
| γ_20_ Intercept | -0.012 | 0.006 | 712 | 1.91 | .057 |
|  |  |  |  |  |  |
|  |  |  |  |  |  |
| Model for the Variance |  | Estimate | SE | *Z*-value | *p*-value |
| Institutional Trust |  |  |  |  |  |
| Overall BP Variance, τ^2^_U10_ | Control | 0.177 | 0.028 | 6.26 | < .001 |
|  | Experimental | 0.300 | 0.063 | 4.74 | < .001 |
|  |  |  |  |  |  |
