# Supplementary material for: A longitudinal and experimental study of the impact of knowledge on the bases of institutional trust
Source: PLoS One. 2017 Apr 17;12(4):e0175387. doi: 10.1371/journal.pone.0175387 (PMC5393579; doi:10.1371/journal.pone.0175387)
Supplement: S2 Table — (DOCX) [file pone.0175387.s007.docx]

S2 Table

*Slopes-as-Outcomes Model 2: Dispositional and Governmental Trust Predicting Trustworthiness.*

| Model Effects | Estimate | SE | *DF* | *t*-value | *p*-value |
| --- | --- | --- | --- | --- | --- |
| Model for the Means  Institutional Trust Intercept (Survey 1 Ratings), β_0_ |  |  |  |  |  |
| γ_00_ Intercept | 4.873 | 0.077 | 88.4 | N/A | N/A |
| γ_01_ Manipulation Effect (0 = Control, 1 = Experimental) | 0.087 | 0.087 | 140 | 1.01 | .316 |
| γ_02_ Dispositional Trust Intercept (0 = mean, 5.436) | 0.456** | 0.145 | 81 | 3.13 | .002 |
| γ_03_ Governmental Trust Intercept (0 = mean, 5.031) | 0.268** | 0.090 | 83.4 | 2.97 | .004 |
| γ_04_ Dispositional Trust Intercept × Manipulation Effect | -0.214 | 0.177 | 161 | 1.21 | .229 |
| γ_05_ Governmental Trust Intercept × Manipulation Effect | -0.022 | 0.120 | 205 | 0.18 | .854 |
| γ_06_ Dispositional Trust Slope | 1.883 | 1.693 | 81 | 1.11 | .270 |
| γ_07_ Governmental Trust Slope | -1.830* | 0.774 | 84.3 | 2.36 | .020 |
| γ_08_ Dispositional Trust Slope × Manipulation Effect | -5.065* | 2.239 | 202 | 2.26 | .025 |
| γ_09_ Governmental Trust Slope × Manipulation Effect | 2.584** | 0.945 | 184 | 2.65 | .009 |
| γ_010_ Dispositional Trust Residual (WP Effect) | 0.200+ | 0.113 | 392 | 1.77 | .077 |
| γ_011_ Governmental Trust Residual (WP Effect) | 0.020 | 0.079 | 45.4 | 0.25 | .806 |
| γ_012_ Dispositional Trust Residual × Manipulation Effect | -0.134 | 0.139 | 572 | 0.97 | .334 |
| γ_013_ Governmental Trust Residual × Manipulation Effect | 0.183+ | 0.099 | 107 | 1.84 | .068 |
|  |  |  |  |  |  |
| Linear Time Slope (0 = Survey 1), β_1_ |  |  |  |  |  |
| γ_10_ Intercept | 0.104*** | 0.028 | 708 | 3.69 | < .001 |
| γ_11_ Manipulation Effect | 0.002 | 0.017 | 687 | 0.10 | .917 |
| γ_12_ Dispositional Trust Intercept | -0.021 | 0.026 | 630 | 0.81 | .417 |
| γ_13_ Governmental Trust Intercept | 0.019 | 0.016 | 560 | 1.17 | .244 |
| γ_14_ Dispositional Trust Intercept × Manipulation Effect | 0.019 | 0.034 | 690 | 0.56 | .578 |
| γ_15_ Governmental Trust Intercept × Manipulation Effect | 0.025 | 0.023 | 696 | 1.10 | .273 |
| γ_16_ Dispositional Trust Slope | 0.888** | 0.302 | 573 | 2.94 | .003 |
| γ_17_ Governmental Trust Slope | 0.413** | 0.139 | 550 | 2.98 | .003 |
| γ_18_ Dispositional Trust Slope × Manipulation Effect | -0.486 | 0.429 | 691 | 1.13 | .258 |
| γ_19_ Governmental Trust Slope × Manipulation Effect | -0.293 | 0.185 | 683 | 1.59 | .113 |
| γ_110_ Dispositional Trust Residual (WP Effect) | -0.012 | 0.032 | 294 | 0.38 | .705 |
| γ_111_ Governmental Trust Residual (WP Effect) | 0.056+ | 0.030 | 123 | 1.87 | .064 |
| γ_112_ Dispositional Trust Residual × Manipulation Effect | 0.021 | 0.043 | 546 | 0.49 | .621 |
| γ_113_ Governmental Trust Residual × Manipulation Effect | -0.064+ | 0.037 | 255 | 1.71 | .089 |
|  |  |  |  |  |  |
| Quadratic Time Slope, β_2_ |  |  |  |  |  |
| γ_20_ Intercept | -0.009+ | 0.005 | 711 | 1.82 | .069 |
|  |  |  |  |  |  |
|  |  |  |  |  |  |
| Model for the Variance |  | Estimate | SE | *Z*-value | *p*-value |
| Institutional Trust |  |  |  |  |  |
| Overall BP Variance, τ^2^_U10_ | Control | 0.241** | 0.049 | 4.87 | < .001 |
|  | Experimental | 0.158** | 0.024 | 6.60 | < .001 |
| Dispositional Trust |  |  |  |  |  |
| Overall BP Variance, τ^2^_U20_ | Control | 0.010 | 0.023 | 0.46 | .323 |
|  | Experimental | 0.030* | 0.015 | 1.99 | .023 |
| Institutional × Dispositional Trust Covariance |  |  |  |  |  |
| Overall BP Covariance, τ^2^_U10_ * τ^2^_U20_ | Control | -0.021 | 0.026 | 0.83 | .405 |
|  | Experimental | 0.026 | 0.016 | 1.64 | .101 |
