# Supplementary material for: A longitudinal and experimental study of the impact of knowledge on the bases of institutional trust
Source: PLoS One. 2017 Apr 17;12(4):e0175387. doi: 10.1371/journal.pone.0175387 (PMC5393579; doi:10.1371/journal.pone.0175387)
Supplement: S3 Table — (DOCX) [file pone.0175387.s008.docx]

S3 Table

*Slopes-as-Outcomes Model 3: Dispositional and Governmental Trust Predicting Distrustworthiness.*

| Model Effects | Estimate | SE | *DF* | *t*-value | *p*-value |
| --- | --- | --- | --- | --- | --- |
| Model for the Means  Institutional Trust Intercept (Survey 1 Ratings), β_0_ |  |  |  |  |  |
| γ_00_ Intercept | 3.759 | 0.074 | 106 | N/A | N/A |
| γ_01_ Manipulation Effect (0 = Control, 1 = Experimental) | -0.125 | 0.093 | 222 | 1.34 | .183 |
| γ_02_ Dispositional Trust Intercept (0 = mean, 5.436) | -0.486** | 0.144 | 103 | 3.39 | .001 |
| γ_03_ Governmental Trust Intercept (0 = mean, 5.031) | -0.055 | 0.089 | 107 | 0.61 | .541 |
| γ_04_ Dispositional Trust Intercept × Manipulation Effect | 0.373 | 0.195 | 250 | 1.91 | .057 |
| γ_05_ Governmental Trust Intercept × Manipulation Effect | -0.199 | 0.137 | 290 | 1.46 | .146 |
| γ_06_ Dispositional Trust Slope | -2.204 | 1.697 | 112 | 1.30 | .197 |
| γ_07_ Governmental Trust Slope | 1.347 | 0.773 | 113 | 1.74 | .084 |
| γ_08_ Dispositional Trust Slope × Manipulation Effect | 4.480 | 2.561 | 304 | 1.75 | .081 |
| γ_09_ Governmental Trust Slope × Manipulation Effect | -1.939 | 1.097 | 286 | 1.77 | .078 |
| γ_010_ Dispositional Trust Residual (WP Effect) | -0.259 | 0.146 | 710 | 1.77 | .077 |
| γ_011_ Governmental Trust Residual (WP Effect) | -0.049 | 0.098 | 709 | 0.50 | .617 |
| γ_012_ Dispositional Trust Residual × Manipulation Effect | 0.209 | 0.177 | 709 | 1.18 | .238 |
| γ_013_ Governmental Trust Residual × Manipulation Effect | 0.027 | 0.120 | 709 | 0.23 | .820 |
|  |  |  |  |  |  |
| Linear Time Slope (0 = Survey 1), β_1_ |  |  |  |  |  |
| γ_10_ Intercept | -0.084*** | 0.018 | 747 | 4.68 | < .001 |
| γ_11_ Manipulation Effect | 0.008 | 0.022 | 746 | 0.39 | .696 |
| γ_12_ Dispositional Trust Intercept | 0.032 | 0.034 | 741 | 0.94 | .349 |
| γ_13_ Governmental Trust Intercept | -0.050* | 0.021 | 736 | 2.38 | .018 |
| γ_14_ Dispositional Trust Intercept × Manipulation Effect | -0.070 | 0.043 | 736 | 1.60 | .110 |
| γ_15_ Governmental Trust Intercept × Manipulation Effect | 0.037 | 0.030 | 732 | 1.26 | .206 |
| γ_16_ Dispositional Trust Slope | -0.429 | 0.392 | 717 | 1.10 | .274 |
| γ_17_ Governmental Trust Slope | -0.159 | 0.179 | 727 | 0.89 | .374 |
| γ_18_ Dispositional Trust Slope × Manipulation Effect | 0.584 | 0.554 | 718 | 1.05 | .293 |
| γ_19_ Governmental Trust Slope × Manipulation Effect | 0.008 | 0.238 | 724 | 0.03 | .973 |
| γ_110_ Dispositional Trust Residual (WP Effect) | 0.080 | 0.041 | 711 | 1.94 | .053 |
| γ_111_ Governmental Trust Residual (WP Effect) | -0.010 | 0.038 | 713 | 0.27 | .790 |
| γ_112_ Dispositional Trust Residual × Manipulation Effect | -0.057 | 0.055 | 711 | 1.04 | .298 |
| γ_113_ Governmental Trust Residual × Manipulation Effect | 0.002 | 0.047 | 712 | 0.05 | .964 |
|  |  |  |  |  |  |
|  |  |  |  |  |  |
| Model for the Variance |  | Estimate | SE | *Z*-value | *p*-value |
| Institutional Trust |  |  |  |  |  |
| Overall BP Variance, τ^2^_U10_ | Control | 0.267*** | .040 | 6.67 | < .001 |
|  | Experimental | 0.179*** | .042 | 4.25 | < .001 |
