# Supplementary material for: A longitudinal and experimental study of the impact of knowledge on the bases of institutional trust
Source: PLoS One. 2017 Apr 17;12(4):e0175387. doi: 10.1371/journal.pone.0175387 (PMC5393579; doi:10.1371/journal.pone.0175387)
Supplement: S4 Table — (DOCX) [file pone.0175387.s009.docx]

S4 Table

*Slopes-as-Outcomes Model 4: Trustworthiness Predicting Institutional Trust.*

| Model Effects | Estimate | SE | *DF* | *t*-value | *p*-value |
| --- | --- | --- | --- | --- | --- |
| Model for the Means  Institutional Trust Intercept (Survey 1 Ratings), β_0_ |  |  |  |  |  |
| γ_00_ Intercept | 5.010 | 0.035 | 126 | N/A | N/A |
| γ_01_ Manipulation Effect (0 = Control, 1 = Experimental) | -0.007 | 0.039 | 195 | 0.18 | .860 |
| γ_02_ Trustworthiness Intercept (0 = mean, 5.171) | 1.114*** | 0.053 | 108 | 20.88 | < .001 |
| γ_03_ Trustworthiness Intercept × Manipulation Effect | -0.037 | 0.065 | 212 | 0.58 | .564 |
| γ_04_ Trustworthiness Slope | -1.336* | 0.515 | 131 | 2.60 | .011 |
| γ_05_ Trustworthiness Slope × Manipulation Effect | 1.333* | 0.626 | 253 | 2.13 | .034 |
| γ_06_ Trustworthiness Residual (WP Effect) | 0.656*** | 0.102 | 112 | 6.45 | < .001 |
| γ_07_ Trustworthiness Residual × Manipulation Effect | 0.460*** | 0.118 | 186 | 3.91 | < .001 |
|  |  |  |  |  |  |
| Linear Time Slope (0 = Survey 1), β_1_ |  |  |  |  |  |
| γ_10_ Intercept | 0.125*** | 0.017 | 720 | 7.46 | < .001 |
| γ_11_ Manipulation Effect | -0.005 | 0.010 | 702 | 0.56 | .577 |
| γ_12_ Trustworthiness Intercept | -0.004 | 0.013 | 658 | 0.33 | .739 |
| γ_13_ Trustworthiness Intercept × Manipulation Effect | 0.003 | 0.016 | 695 | 0.19 | .846 |
| γ_14_ Trustworthiness Slope | 1.478*** | 0.124 | 508 | 11.94 | < .001 |
| γ_15_ Trustworthiness Slope × Manipulation Effect | -0.432** | 0.155 | 664 | 2.78 | .006 |
| γ_16_ Trustworthiness Residual (WP Effect) | 0.071* | 0.033 | 161 | 2.12 | .036 |
| γ_17_ Trustworthiness Residual × Manipulation Effect | -0.120** | 0.040 | 292 | 3.00 | .003 |
|  |  |  |  |  |  |
| Quadratic Time Slope, β_2_ |  |  |  |  |  |
| γ_20_ Intercept | -0.014*** | 0.003 | 718 | 4.77 | < .001 |
|  |  |  |  |  |  |
|  |  |  |  |  |  |
| Model for the Variance |  | Estimate | SE | *Z*-value | *p*-value |
| Institutional Trust |  |  |  |  |  |
| Overall BP Variance, τ^2^_U10_ | Control | 0.039 | 0.009 | 4.40 | < .001 |
|  | Experimental | 0.024 | 0.004 | 5.55 | < .001 |
| Trustworthiness |  |  |  |  |  |
| Overall BP Variance, τ^2^_U20_ | Control | 0.072 | 0.037 | 1.93 | .027 |
|  | Experimental | 0.042 | 0.020 | 2.12 | .017 |
| Institutional × Trustworthiness Covariance |  |  |  |  |  |
| Overall BP Covariance, τ^2^_U10_ * τ^2^_U20_ | Control | -0.033 | 0.016 | 2.03 | .042 |
|  | Experimental | -0.008 | 0.008 | 1.06 | .290 |
