# Supplementary material for: A longitudinal and experimental study of the impact of knowledge on the bases of institutional trust
Source: PLoS One. 2017 Apr 17;12(4):e0175387. doi: 10.1371/journal.pone.0175387 (PMC5393579; doi:10.1371/journal.pone.0175387)
Supplement: S5 Table — (DOCX) [file pone.0175387.s010.docx]

S5 Table

*Slopes-as-Outcomes Model 4: Distrustworthiness Predicting Institutional Trust.*

| Model Effects | Estimate | SE | *DF* | *t*-value | *p*-value |
| --- | --- | --- | --- | --- | --- |
| Model for the Means  Institutional Trust Intercept (Survey 1 Ratings), β_0_ |  |  |  |  |  |
| γ_00_ Intercept | 4.997 | 0.065 | 111 | N/A | N/A |
| γ_01_ Manipulation Effect (0 = Control, 1 = Experimental) | -0.010 | 0.077 | 190 | 0.13 | .894 |
| γ_02_ Distrustworthiness Intercept (0 = mean, 5.171) | -1.189*** | 0.140 | 83.3 | 8.51 | < .001 |
| γ_03_ Distrustworthiness Intercept × Manipulation Effect | 0.227 | 0.163 | 147 | 1.40 | .165 |
| γ_04_ Distrustworthiness Slope | -0.856 | 0.781 | 73.3 | 1.10 | .277 |
| γ_05_ Distrustworthiness Slope × Manipulation Effect | -0.630 | 0.928 | 138 | 0.68 | .499 |
| γ_06_ Distrustworthiness Residual (WP Effect) | -0.137 | 0.169 | 143 | 0.81 | .419 |
| γ_07_ Distrustworthiness Residual × Manipulation Effect | -0.315 | 0.196 | 236 | 1.61 | .109 |
|  |  |  |  |  |  |
| Linear Time Slope (0 = Survey 1), β_1_ |  |  |  |  |  |
| γ_10_ Intercept | 0.083** | 0.031 | 688 | 2.69 | .007 |
| γ_11_ Manipulation Effect | 0.021 | 0.018 | 674 | 1.19 | .236 |
| γ_12_ Distrustworthiness Intercept | -0.011 | 0.038 | 635 | 0.29 | .771 |
| γ_13_ Distrustworthiness Intercept × Manipulation Effect | 0.058 | 0.043 | 679 | 1.35 | .178 |
| γ_14_ Distrustworthiness Slope | -0.594** | 0.204 | 591 | 2.91 | .004 |
| γ_15_ Distrustworthiness Slope × Manipulation Effect | 0.312 | 0.237 | 653 | 1.32 | .188 |
| γ_16_ Distrustworthiness Residual (WP Effect) | -0.062 | 0.051 | 348 | 1.21 | .226 |
| γ_17_ Distrustworthiness Residual × Manipulation Effect | 0.095 | 0.061 | 506 | 1.57 | .118 |
|  |  |  |  |  |  |
| Quadratic Time Slope, β_2_ |  |  |  |  |  |
| γ_20_ Intercept | -0.010+ | 0.006 | 691 | 1.72 | .086 |
|  |  |  |  |  |  |
|  |  |  |  |  |  |
| Model for the Variance |  | Estimate | SE | *Z*-value | *p*-value |
| Institutional Trust |  |  |  |  |  |
| Overall BP Variance, τ^2^_U10_ | Control | 0.150 | 0.035 | 4.33 | < .001 |
|  | Experimental | 0.136 | 0.022 | 6.22 | < .001 |
| Distrustworthiness |  |  |  |  |  |
| Overall BP Variance, τ^2^_U20_ | Control | 0.316 | 0.117 | 2.71 | .003 |
|  | Experimental | 0.230 | 0.070 | 3.31 | < .001 |
| Institutional × Distrustworthiness Covariance |  |  |  |  |  |
| Overall BP Covariance, τ^2^_U10_ * τ^2^_U20_ | Control | 0.157 | 0.058 | 2.69 | .007 |
|  | Experimental | 0.079 | 0.028 | 2.85 | .004 |
